# Bayesian model selection validates a biokinetic model for zirconium processing in humans

**DOI:** 10.1186/1752-0509-6-95

**Published:** 2012-08-05

**Authors:** Daniel Schmidl, Sabine Hug, Wei Bo Li, Matthias B Greiter, Fabian J Theis

**Affiliations:** 1 Institute of Bioinformatics and Systems Biology, Helmholtz Zentrum München German Research Center for Environmental Health, Neuherberg, Germany; 2 Institute for Mathematical Sciences, Technische Universität München, Garching, Germany; 3 Research Unit Medical Radiation Physics and Diagnostics, Helmholtz Zentrum München German Research Center for Environmental Health, Neuherberg, Germany

**Keywords:** Bayesian inference, Model selection, MCMC sampling, Compartmental model, Internal dosimetry, Systems biology

## Abstract

**Background:**

In radiation protection, biokinetic models for zirconium processing are of crucial importance in dose estimation and further risk analysis for humans exposed to this radioactive substance. They provide limiting values of detrimental effects and build the basis for applications in internal dosimetry, the prediction for radioactive zirconium retention in various organs as well as retrospective dosimetry. Multi-compartmental models are the tool of choice for simulating the processing of zirconium. Although easily interpretable, determining the exact compartment structure and interaction mechanisms is generally daunting. In the context of observing the dynamics of multiple compartments, Bayesian methods provide efficient tools for model inference and selection.

**Results:**

We are the first to apply a Markov chain Monte Carlo approach to compute Bayes factors for the evaluation of two competing models for zirconium processing in the human body after ingestion. Based on *in vivo* measurements of human plasma and urine levels we were able to show that a recently published model is superior to the standard model of the International Commission on Radiological Protection. The Bayes factors were estimated by means of the numerically stable *thermodynamic integration* in combination with a recently developed copula-based Metropolis-Hastings sampler.

**Conclusions:**

In contrast to the standard model the novel model predicts lower accretion of zirconium in bones. This results in lower levels of noxious doses for exposed individuals. Moreover, the Bayesian approach allows for retrospective dose assessment, including credible intervals for the initially ingested zirconium, in a significantly more reliable fashion than previously possible. All methods presented here are readily applicable to many modeling tasks in systems biology.

## Background

Radioactive zirconium (Zr) isotopes are produced in large quantities in nuclear fission reactors; one of the most common fragments in uranium fission is the radioactive ^95^Zr. For example, the estimated released ^95^Zr activity of the Fukushima and Chernobyl accidents is considered to have detrimental health effects not only via irradiation, but also via the intake of edibles [1,2]. The estimation of radiation doses is indispensable for risk analysis. This is true for occupational exposure [3] and patients undergoing diagnostic and therapeutic nuclear medicine [4] as well as for the public in general [5]. To calculate the radiation dose a mathematical model is required for quantifying the deposition of radioactivity from the incorporated radionuclide inside the human body. In internal dosimetry, this model is called biokinetic model as defined by the International Commission on Radiological Protection (ICRP) in [5]. Also, the ICRP put forward the current standard model, which we will simply denote the ICRP model. The parameters of this model were mostly derived from animal data. In order to obtain more reliable dose estimates for humans, the Helmholtz Zentrum München (HMGU) developed a new, physiologically more plausible biokinetic model [6]. It is based on the processing of non-radioactive Zr isotopes in 16 investigations with 12 healthy human subjects. The measurements were taken *in vivo* in plasma and urine up to 100 days after administration by application of the double tracer technique. Moreover, a global statistical analysis method has been developed and the uncertainty and sensitivity of the HMGU model parameters were analyzed [7,8].

The biokinetic models mentioned above incorporate basic processes in the human physiological system [3,5,9,10]. Mathematically, this is characterized as follows: All major human organs and tissues are simplified in the model structure as separate compartments that represent kinetically homogeneous amounts of radionuclides; the connections between organs and tissues are described via transfer rates, i.e. model parameters that represent the exchange rates between the individual mutually exclusive compartments. These multi-compartmental systems along with their transfer parameters describing the kinetic behavior of radionuclides in the human body are called *compartmental models*[5,11]. Throughout this paper we use the terms biokinetic model and compartmental model interchangeably. The transfer of substances into and out of compartments is governed by the law of mass balance. Transfer parameters are frequently evaluated on the basis of experimental data obtained from laboratory animals and, to a lesser extent, human beings [10]. Although animal data is not directly comparable to human data, the former can nevertheless be very helpful as prior information.

In this publication, we address the problem of model inference and model selection. A Bayesian approach enables us to cover model and measurement uncertainties for a compartmental model based on human data, while simultaneously taking into account the prior information. The Bayesian framework provides a fully probabilistic approach [12]. It is grounded on the probability distribution of a problem specific parameter space conditioned on the given data. This specifies a measure of belief for all possible parameter values. Similarly – albeit not identical – to confidence intervals, Bayesian analyses provide credible sets for the parameters at stake, holding regions and limits of high parameter probability [13]. However, as they are intrinsically driven by prior informations, some care has to be taken in their interpretation [14].

For an overall assessment of the two competing biokinetic models for Zr, the previous model parameter uncertainty analysis [7,8] is not sufficient, because uncertainties arising from the model structure were not taken into account. This shortcoming was addressed by our Bayesian approach. Considering the models themselves as a random variable allows to compute the probability for each of the models conditioned on given data. The ratio of the marginal likelihoods of two models, i.e. the ratio of the probability for the data to be produced by the specific model, is known as the *Bayes factor*, a quantity introduced by Jeffreys [15]. Performing model selection using Bayes factors is particularly useful when dealing with a few models only. While classical model selection approaches using statistics such as the AIC or likelihood ratio tests are based on single best parameter estimates [16], the Bayes factor takes into account all possible parameters values and thus deals with model uncertainty and avoids overfitting issues [17,18]. Moreover, in contrast to classical tests, the Bayes factor provides evidence for either one of the (possibly non-nested) models by definition. With the introduction of Markov chain Monte Carlo (MCMC) methods [19-21] as tools for sampling from probability distributions, a remarkable increase in Bayesian analyses was noticed. However, due to very complex probability surfaces these approaches often struggle with sampling efficiency [22]. In order to avoid resulting convergence issues of the MCMC approach, we combined a technique called *thermodynamic integration* with a novel copula-based Metropolis-Hastings sampler [23]. This provides numerically stable results for the inference process.

The HMGU and ICRP models were compared based on *in vivo* plasma and urine data of 16 investigations of 12 human subjects [6] using Bayes factors. More precisely, the models were evaluated for each investigation individually and for the concatenated data of all investigations. The latter allows to infer transfer rates (including credible intervals) for an average individual. We furthermore provide an analysis based on the (i) plasma data and (ii) urine data individually. Throughout the analysis, the HMGU model turned out to be superior compared to the ICRP model with respect to covering the specific data. This means the HMGU model more precisely explains the given observations and therefore the processing of zirconium in the human body. We then used the HMGU model to analyze the accretion of zirconium in bones: not only did we observe a delayed aggregation but also to lesser extents than predicted by the ICRP model. Lastly, the Bayesian framework yielded credible bounds for retrospective dose assessment of an average individual, this is, based on the concatenated data of all 16 investigations. We provide a user-friendly estimation table for the prediction of initially ingested zirconium concentrations for *ex post* measurements. This impacts the estimation of intake and radiation dose, especially the bone dose, when aiming for personalized occupational monitoring data.

## Methods

### Biokinetic models for zirconium processing

**Figure 1:**
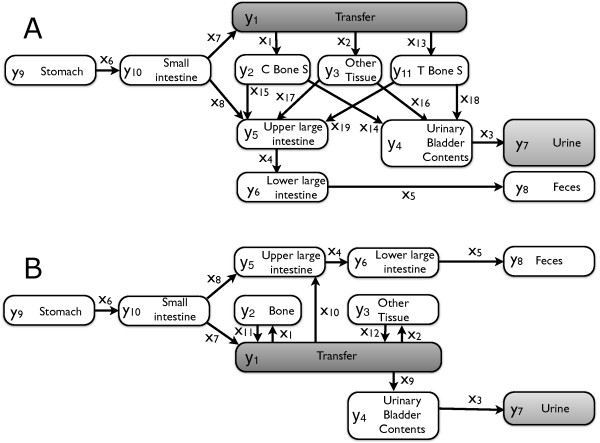
**Models for the biokinetics of zirconium.A**: ICRP model. The model consists of eleven compartments *y*_1_,…,*y*_11_and 15 time independent transfer rates *x*_1_,…,*x*_8_,*x*_13_,…,*x*_19_. **B**: HMGU model. The model consists of ten compartments *y*_1_,…,*y*_10_and twelve transfer rates *x*_1_,…,*x*_12_. In both models zirconium enters the body in the stomach compartment *y*_9_and diffuses through the system until it reaches either one of the two final compartments urine, *y*_7_, or feces, *y*_8_. The gray compartments *y*_1_and *y*_7_are directly related to the datasets measured.

We infer the biokinetics of zirconium as it is processed in the human body. The currently used compartmental model was recommended by the ICRP in [5,10,24] (Figure 1A). It consists of eleven compartments and 15 transfer rates. Zirconium enters the body via the stomach compartment *y*_9_and is processed until it reaches any of the two final compartments urine, *y*_7_, or feces, *y*_8_. Some of the transfer rates and compartments of the ICRP model are however physiologically questionable: The direct mass transport from the two bone compartments to the urinary bladder contents and upper large intestine compartments or the distinction between trabecular bone surface and cortical bone surface as such. In order to address these shortcomings, we have recently proposed the alternative HMGU model [6] combining the two bone compartments into one single compartment and replacing these mass flows by physiologically more plausible transfer rates (Figure 1B). Altogether both models share eight transfer rates, which we denoted by *x*_1_,…,*x*_8_. Transfers present in just one of the models have a unique index to facilitate distinction.

The dynamics of both models are described by a system of coupled linear first-order ordinary differential equations (ODEs): For each compartment *y*_*j*_ with time-dependent concentration *y*_*j*_(*t*), the rate of change of *y*_*j*_is given by

(1) $$\frac{\text{d}}{\text{d}t}y_{j}(t)=\underset{\alpha \in A_{y_{j}}^{+}}{\sum }x_{\alpha }y_{\left[x_{\alpha }\right]}(t)-\underset{\beta \in A_{y_{j}}^{-}}{\sum }x_{\beta }y_{j}(t)$$

where $A_{y_{j}}^{+}$ is the set of indices of all transfer rates *x*_*α*_flowing into compartment *y*_*j*_, $A_{y_{j}}^{-}$ the set of indices of all transfer rates flowing out of compartment *y*_*j*_and $y_{\left[x_{\alpha }\right]}$ is the compartment which is connected to *y*_*j*_by the transfer rate *x*_*α*_. For instance $A_{y_{5}}^{+}=\{8,10\}$, $y_{\left[x_{8}\right]}=y_{10}$ and $y_{\left[x_{10}\right]}=y_{1}$ in the HMGU model. We have *y*_9_(0)=100*%*and therefore *y*_*j*≠9_(0)=0*%*, this is, the complete amount of zirconium is initially contained in the stomach compartment. The explicit model specific ODE systems can be found in the Additional file 1 sections 1.1 and 1.2.

### Experimental data

The human biokinetic data was measured in a stable tracer study executed at the Helmholtz Zentrum München (HMGU) [6,25]. It includes 16 investigations of 12 healthy humans with ingestion of a investigation-specific amount of isotopically enriched stable zirconium. The administered amount was based on the individuals weight, aiming at a dose of 0.09mg stable tracer per kg body weight. Tracer concentrations were determined in blood plasma and urine. For the plasma data, samples were taken several times during the first day in increasing intervals, and more scarcely later on. Urine was collected completely in 12-24h periods on several days. The last samples were taken at 100d after tracer administration. Tracer concentrations were normalized to the respective tracer amount ingested in each investigation, such that the total ingested amount corresponds to 100*%* at *t*=0 in the stomach compartment *y*_9_. For model development, the transfer compartment was taken to be identical with blood plasma, and concentrations therein were expressed as % per kg plasma. The plasma concentration was scaled by the total amount of plasma in the body to get absolute concentrations [26]. Urine data was expressed as excretion rate in % per day.

### Model likelihood

Technical limitations, such as missing *in vivo* measurement techniques for all involved compartments as well as noisy data introduce model uncertainties to biological systems [27]. Comparing different kinds of models based on single parameter estimates as done in maximum-likelihood approaches is thus very critical. For statistical model evaluation we here applied a Bayesian approach, taking into account the full parameter distribution: For each investigation *i* we assume that the data

$$D_{i}=(y_{1}^{i,1},y_{1}^{i,2},\ldots ,y_{1}^{i,n_{i}^{b}},{\dot{y}}_{7}^{i,1},{\dot{y}}_{7}^{i,2},\ldots ,{\dot{y}}_{7}^{i,n_{i}^{u}})$$

follows the solution $c_{x^{k}}(t)$ of the differential equation given in (1) for any of the two models $M^{k}$ and some corresponding parameter vector ***x***^*k*^, where the model index *k*∈{*HI*}. Here, $M^{I}$ is the ICRP model and $M^{H}$ the HMGU model. Corresponding to the notation in Figure 1A and 1B, $x^{I}=(x_{1},\ldots ,x_{8},x_{13},\ldots ,x_{19})$ and $x^{H}=(x_{1},\ldots ,x_{12})$. While $y_{1}^{i,\cdot }$ indicates measurements in plasma, i.e. in the transfer compartment *y*_1_, ${\dot{y}}_{7}^{i,\cdot }$ designates measurements of the excretion rate in the urine compartment *y*_7_. There are $n_{i}^{b}$ measurements in plasma and $n_{i}^{u}$ measurements in urine for investigation *i*. Assuming furthermore that all data points contain normally distributed measurement errors, the investigation *i* and model *k* specific likelihood function has the form

$$\begin{array}{c} L_{i}(x^{k},k|D_{i})=\underset{L_{i}^{b}(x^{k},k|D_{i})}{\underset{︸}{\overset{n_{i}^{b}}{\underset{\alpha =1}{\prod }}\Phi \left(\right)y_{1}^{i,\alpha }|c_{x^{k}}^{b}(t_{\alpha }),\sigma_{i}^{b}\left(\right)}}\\ \;\times \underset{L_{i}^{u}(x^{k},k|D_{i})}{\underset{︸}{\overset{n_{i}^{u}}{\underset{\beta =1}{\prod }}\Phi \left({\dot{y}}_{7}^{i,\beta }|\frac{d}{\mathrm{dt}}c_{x^{k}}^{u}(t_{\beta }),\sigma_{i}^{u}\right)}}, \end{array}$$

where $c_{x^{k}}^{b}(t_{\alpha })$ denotes the solution to Equation (1) for the transfer compartment *y*_1_at time point *t*_*α*_, corresponding to the measurement at $\left(y_{1}^{i,\alpha }\right)$, for the parameter vector ***x***^*k*^. Furthermore, $\frac{d}{\mathrm{dt}}c_{x^{k}}^{u}(t_{\beta })$ is the derivative of the solution for the urine compartment *y*_7_at time point *t*_*β*_, corresponding to the measurement ${\dot{y}}_{7}^{i,\beta }$, while *Φ*(·|*μσ*) is the univariate probability density function of the normal distribution with mean *μ* and standard deviation *σ*.

The standard deviations for plasma, $\sigma_{i}^{b}$, and for urine, $\sigma_{i}^{u}$, are fitted investigation-specifically by simulated annealing [28] before starting the MCMC sampling process. They correspond to the combined strength of all deviations from the ODE, e.g. to the size of the measurement error as well as to the magnitude of the difference between the ODE solution and the data points that is due to natural internal fluctuations not considered by an ODE approach. The biological variability between the individual investigations is accounted for by the fact that we fit different $\sigma_{i}^{b}$ and $\sigma_{i}^{u}$ for each investigation i and thus get investigation-specific likelihoods. This leads to individual credible intervals for each parameter in each investigation in the MCMC sampling procedure later on.

The complete data (i.e. concatenated data) likelihood is given by $L_{\mathrm{ALL}}(x^{k},k|D)=\overset{16}{\underset{i=1}{\prod }}L_{i}(x^{k},k|D_{i})$ for the complete data $D=\{D_{1},\ldots ,D_{16}\}$ where in all $L_{i}(x^{k},k|D_{i})$ the same fitted investigation independent $\sigma_{i}^{b}=\sigma^{b}$ and $\sigma_{i}^{u}=\sigma^{u}$ are used.

For the calculation of $L_{i}(x^{k},k|D_{i})$ Equation (1) has to be solved based on ***x***^*k*^. Since (1) is of first order, it can be written as

(2) $$\frac{dy_{x^{k}}(t)}{\mathrm{dt}}=A(x^{k})\cdot y_{x^{k}}(t),$$

where $y_{x^{k}}(t)$ is the vector of all the compartments of model *k* and the time independent matrix *A*(***x***^*k*^) represents all the interactions between these compartments, depending on the transfer rate values ***x***^*k*^. The analytical solution is then given by

(3) $$y_{x^{k}}(t)=e^{A(x^{k})t}\cdot y_{x^{k}}(t=0).$$

The matrix exponential *e*^*A*(^***x****k*^)*t*^was computed by eigenvalue decomposition using MATLAB’s eig function (see Additional file 1 section 1.3).

### Bayes factors

Specifying model specific, investigation independent prior distributions *p*(***x***^*k*^|*k*) based on combined human/animal data yields the posterior distributions of the parameters for model *k* according to Bayes’ theorem:

(4) $$p(x^{k}|D_{i},k)=\frac{L_{i}(x^{k},k|D_{i})p(x^{k}|k)}{p(D_{i}|k)},$$

where $p(D_{i}|k)$ denotes the marginal density obtained by integrating over the according parameter space ***X***^*k*^, i.e.

(5) $$p(D_{i}|k)=\underset{X^{k}}{\int }L_{i}(x^{k},k|D_{i})p(x^{k}|k)\;\text{d}x^{k}.$$

The quantity $p(D_{i}|k)$ is called data evidence and is the basis for the model selection process. For comparing two models *k* and *k*^*′*^, we can view the model index as a random variable, apply Bayes’ theorem again and take ratios to cancel the denominator, yielding

(6) $$\frac{p(k|D_{i})}{p(k^{\prime }|D_{i})}=\frac{p(D_{i}|k)}{p(D_{i}|k^{\prime })}\cdot \frac{p(k)}{p(k^{\prime })}.$$

The ratio of the two likelihoods in Equation (6) is known as the *Bayes factor*

(7) $$B_{k,k^{\prime }}^{i}=\frac{p(D_{i}|k)}{p(D_{i}|k^{\prime })}.$$

We had no initial preference for any of the models and thus chose a uniform model prior. The Bayes factor in Equation (7) then coincides with the posterior odds ratio between the models *k* and *k*^*′*^ conditioned on the data $D_{i}$[18,29].

The Bayes factor compares the posterior probability $p(k|D_{i})$ that the data $D_{i}$ have arisen according to model *k* versus the probability $p(k^{\prime }|D_{i})=1-p(k|D_{i})$ that $D_{i}$ have arisen according to model *k*^*′*^, in contrast to single parameter measures such as the AIC or the likelihood ratio test [16]. The models may be non-nested. Due to the evaluation of each model on its whole parameter space ***X***^*k*^(cf. Equation (5)), the Bayes factor naturally penalizes model complexity and thus prevents overfitting issues [30-32]. An interpretation of the likelihood ratio in the Bayes factor was given by Jeffreys [15], which can also be found in the Additional file 1 section 3. Most importantly, a Bayes factor $B_{k,k^{\prime }}^{i}>3$*substantially* favors model *k*, while $B_{k,k^{\prime }}^{i}>100$*decisively* favors model *k*. Clearly, for $B_{k,k^{\prime }}^{i}<1$, model *k*^*′*^ is favored with evidence $B_{k^{\prime },k}^{i}=1/B_{k,k^{\prime }}^{i}$.

### Prior information

Since the problem of radiation protection has been extensively researched, quite a large number of animal studies have been conducted. These yielded excessive prior knowledge for the Bayesian modeling approach. As the ICRP model is the recommended model used for dose estimation, there naturally exists information on the distribution types of the parameters involved in the model together with confidence intervals [7]. Four parameters have a lognormal distribution, five a triangular distribution and six have a normal distribution (see Additional file 1 section 2.3 for details). From the confidence intervals, the parameters of the normal and lognormal distributions were calculated. For the HMGU model detailed prior information is also available from previous studies [7,8]. Here, eight parameters have a lognormal distribution and four a triangular one (see Additional file 1 section 2.3 for details). It is noteworthy that of the eight parameters shared in both models, *x*_8_ is the only one having different distributions in the ICRP and HMGU model. Due to a lack of prior knowledge of the dependency structure between the parameters, the multivariate prior distribution *p*(***x***^*k*^|*k*) of model *k* was taken to be the product of the univariate prior distributions $p(x_{r}^{k}|k)$ for each parameter $x_{r}^{k}$, i.e. $p(x^{k}|k)=\underset{r}{\prod }p(x_{r}^{k}|k)$. Each univariate prior distribution was truncated at zero. While Bayes factors were computed *inter alia* for each investigation separately (see Results and discussion), the same prior information was applied throughout all investigations. This is reasonable since the priors contain information from a huge variety of preceding experiments.

### Thermodynamic integration

Computing the marginal of Equation (5) by numerical integration generally turns out to be a daunting task. There exist a variety of approaches to tackle this problem. Popular choices include Chib’s method, which is based on a Gibbs sampling scheme and therefore not always easily applicable [33] or the Reversible Jump MCMC algorithm proposed by Green [34], based on an across model search. Since the latter involves sampling of an additional model parameter, the sampling generally takes longer than sampling within the different models only. We therefore applied a within model sampling technique called *thermodynamic integration* (TI). It was proposed by Lartillot and Philippe [35] and Friel and Pettitt [36] and successfully applied e.g. in Xu *et al.*[37]. The method splits apart the computation into several intermediate steps by introducing an auxiliary “temperature” parameter *τ*∈[0,1] that governs the influence of the parameter likelihood. The basis of this approach is the *power posterior*, which is the usual posterior modified such that the likelihood is exponentiated by the temperature parameter and then normalized accordingly to obtain a probability density:

(8) $$p_{\tau }(D_{i}|k)=\frac{L_{i}{\left(x^{k},k|D_{i}\right)}^{\tau }p(x^{k}|k)}{z(D_{i}|k,\tau )}.$$

More precisely, the quantity of interest is the normalization constant

(9) $$z(D_{i}|k,\tau )=\underset{X^{k}}{\int }L_{i}{\left(x^{k},k|D_{i}\right)}^{\tau }p(x^{k}|k)\;\text{d}x^{k}$$

since it yields a way to compute the terms of the Bayes factor (cf. Equation (7)) by differentiating its logarithm

(10) $$\begin{array}{lll} \frac{d}{\mathrm{d\tau}}\log z(D_{i}|k,\tau ) & =\underset{X^{k}}{\int }\log L_{i}(x^{k},k|D_{i})\; & \;\\ & \times \frac{L_{i}{\left(x^{k},k|D_{i}\right)}^{\tau }p(x^{k}|k)}{z(D_{i}|k,\tau )}\;\text{d}x^{k}\; & \;\\ & =E_{p_{\tau }}\left[\log L_{i}(x^{k},k|D_{i})\right]\; \end{array}$$

and then integrating both sides with respect to *τ*

(11) $$\log(p(D_{i}|k))=\overset{1}{\underset{0}{\int }}E_{p_{\tau }}\left[\log L_{i}(x^{k},k|D_{i})\right]\;\text{d}\tau ,$$

according to Calderhead and Girolami [38]. This means that the natural logarithm of the marginal likelihood can be computed as the integral over the expectation of the logarithmized data likelihood within the model with respect to the power posterior. The parameter *τ*governs the flatness of the power posterior surface and, much as in the concept of path sampling [39], stabilizes the computation of Equation (5) [36]: choosing a discretization 0=*τ*_1_<*τ*_2_<…<*τ*_*N*−1_<*τ*_*N*_=1, we can compute the natural logarithm of the marginal likelihood $p(D_{i}|k)$ by numerically approximating the integral in Equation (11) by

(12) $$\begin{array}{c} \;\log(p(D_{i}|k))\approx \overset{N-1}{\underset{n=1}{\sum }}\frac{1}{2}(\tau_{n+1}-\tau_{n})\{E_{p_{\tau_{n}+1}}\left[\log L_{i}(x^{k},k|D_{i})\right]\\ \;+E_{p_{\tau_{n}}}\left[\log L_{i}(x^{k},k|D_{i})\right]\}. \end{array}$$

Also, the expectation with respect to the power posterior in Equation (12) is approximated in the usual way by substituting it with the Monte Carlo estimate

(13) $$E_{p_{\tau_{n}}}\left[\log L_{i}(x^{k},k|D_{i})\right]\approx \frac{1}{M}\overset{M}{\underset{m=1}{\sum }}\log L_{i}\left(x_{\left(m\right)}^{k},k|D_{i}\right),$$

where $x_{\left(m\right)}^{k}$ denotes a sample drawn from $p_{\tau_{n}}(D_{i}|k)$. For all our applications we chose a temperature schedule with *N*=30 steps according to $\tau_{n}={\left(\frac{n-1}{N-1}\right)}^{5},n=1,\ldots ,N$ to estimate $\log(p(D_{i}|k))$ for each *k* and *i* as suggested by Calderhead and Girolami [38].

### Copula-based Monte Carlo sampling

The model, investigation, and temperature specific underlying Markov chain Monte Carlo (MCMC) samples were drawn using the recently introduced copula-based Metropolis-Hastings (MH) algorithm [23]. Copulas are constructs from probability theory for assessing and sampling from multivariate distributions. They are widely used in finance and ecology [40,41]. For any absolutely continuous multivariate cumulative distribution function (cdf) *F*(*x*_1_,…,*x*_*d*_) with marginal cdf’s *F*_*i*_(*x*_*i*_),*i*=1,…,*d*, joint density function *f*(*x*_1_,…,*x*_*d*_) and marginal density functions *f*_*i*_(*x*_*i*_),*i*=1,…,*d*, we decompose

(14) $$f(x_{1},\ldots ,x_{d})=c\left(F_{1}(x_{1}),\ldots ,F_{d}(x_{d})\right)\cdot f_{1}(x_{1})\cdot \ldots \cdot f_{d}(x_{d}),$$

where $c\left(u_{1},\ldots ,u_{d}\right)$ is a unique copula density function defined on [0,1]^*d*^ with uniformly distributed marginals on [0,1]. This copula function covers the full dependency structure of the variables. In other words, every joint distribution function can be decomposed into the marginal behavior of its individual variables and a function covering its dependency structure [42]. The MH proposal function then generates problem specific proposals with an according dependence structure drawn from a pair copula distribution. Fitting the copula distribution was done in preruns containing 1,000,000 unthinned samples each. They were generated for each investigation and model separately. For back-and-forth conversion of the prerun samples and proposals [23], we naturally applied the according prior distributions of the models at hand. Choosing different conversion functions is possible, but affects the sampling performance. Before starting the final MCMC sampling procedure, the maximum a posteriori parameter estimates were computed by simulated annealing and used as initial MCMC sampling values. This makes a burn-in period dispensable. For thinning the Markov chains, i.e. for drawing approximately independent samples in the MCMC procedure, we applied the autocorrelation-based Effective Sample Size (ESS) proposed by Kass [43]. The ESS holds the number of samples left when the Markov chain is thinned such that two consecutive samples can be considered approximately independent. The copula-based MH approach is able to deal with the dependence structure in the high dimensional sampling space and allows for high proposal acceptance rates at simultaneously high ESS’s. Finally, all Bayes factors were computed based on 30,000 proposals of the copula-based MH algorithm at each *τ*_*n*_throughout all applications.

## Results and discussion

In this section, we present the results of our analysis. We address the question which model is superiorly fitting the data. First, several general results, such as investigation dependency of the Bayes factor and effects of parameter correlations are shown, before turning to the results of the model selection, and their consequences for the HMGU and ICRP models.

### Investigation specificity of transfer rates

**Figure 2:**
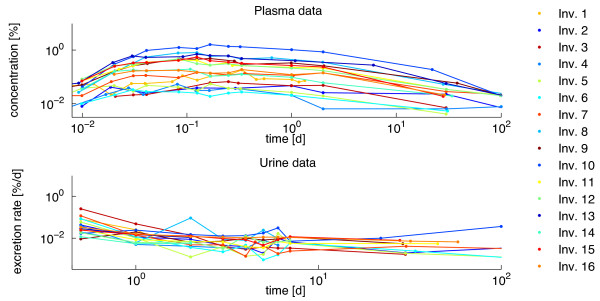
**The experimental data.** Plasma and urine data for investigations 1-16 on log-log-timescale.

In radiation protection the transfer rates for the biokinetics of radionuclides in the human body are derived from data collected in various independent experiments [5]. We measured plasma and urine levels in 16 different investigations. This poses the question whether the models should be compared based on the complete dataset, or whether statistical evaluation should be done for each investigation individually. While the former approach results in one overall Bayes factor, the latter yields 16 investigation specific, not directly comparable Bayes factors. All investigations follow the same pulse-like time courses in the transfer compartment *y*_1_ while the excretion rates in the urine compartment *y*_7_exhibit an exponential decay behavior (Figure 2). However, zirconium tracer concentrations showed up to a 50-fold difference between maximal plasma concentrations, i.e. for investigation 10 (1.616*%*) and 6 (0.033*%*).

To test the hypothesis whether the diversity in concentration also effects transfer rates and therefore the estimated Bayes factors, we pairwise compared the posterior sample marginals of the MCMC run (corresponding to the samples of *τ*=1) for parameter *x*_7_of the ICRP model between all investigations by means of a Kolmogorov-Smirnov test. Here *x*_7_was chosen as it directly affects the observed plasma levels [8]. Except for one pair, all p-values were <6·10^−8^, meaning that the chance of falsely rejecting the hypothesis of comparable marginals is negligible. Therefore, as the posterior marginal distributions are quite different, it can be deduced that the basis for the Bayes factor, the joint posterior distribution, can differ quite strongly w.r.t. the individuals. This indicated that each investigation should be treated separately. Nevertheless, in order to infer the transfer rates of an average subject, as currently used by the ICRP, the concatenated data had to be used. We therefore compared the HMGU and ICRP model based on both the concatenated data $D=\{D_{1},\ldots ,D_{16}\}$ and, in order to account for the biological diversity, the individual investigation specific datasets $D_{i}$ (*i*=1,…,16). This could also be the basis for further analysis of influence factors such as weight or gender.

### MCMC-based parameter estimation

Throughout, the analysis was based on 30,000 proposals for each of the 30 temperature levels in all 17 cases (one for each investigation and one for n). For the HMGU model the average ESS including one standard error, i.e. taken over all temperature levels and investigations, is 5832±405. In case of the ICRP model we obtained in average 5808±252 (approximately independent) samples from the Markov chains. This naturally implied high acceptance rates for both models. The sampling procedure thus captured the power posteriors very well.

**Figure 3:**
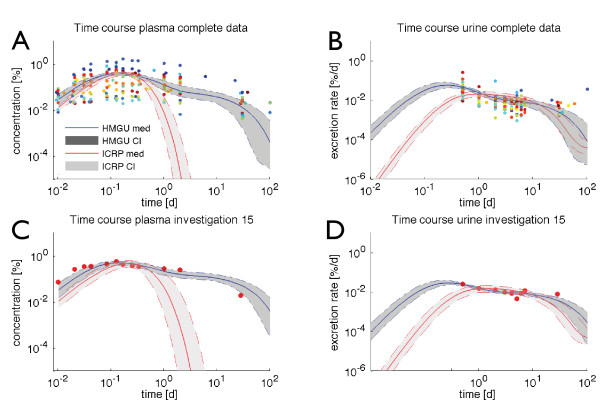
**Posterior time courses.** Sample median (solid line) and 90% credible interval (CI, shaded area) for the numerical solution of the time courses based on the *τ*=1 HMGU (blue) and ICRP (red) MCMC samples for the complete plasma data (**A**), urinary excretion rate over time of the complete data (**B**), plasma data of exemplary investigation 15 (**C**), and urinary excretion rate over time of exemplary investigation 15 (**D**) on a log-log scale. The median and CI represent the uncertainty in the parameters, in contrast to measurement uncertainty (not shown). Colored markers are the data points. The median and the 90% credible interval were computed pointwisely at each time point over all MCMC-based solutions. For readability we truncated plasma plots at 1·10^−5^[*%*] and urine plots at 1·10^−6^[*%*/*d*].

From the posterior samples, we could derive new credible intervals for the parameters at hand as well as a new MAP estimate for an average subject which can be used if single parameter values are required (see Additional file 1 section 4.1). As can be seen in Figure 3, the fit of the time courses covered the data, indicating that both models are in principle able to fit the data. This justifies our ODE approach with additive noise. However, from the fits alone it is not obvious which model is superior. Note that the credible intervals in Figure 3 represent only the uncertainty based on the parameters, in contrast to measurement uncertainties accounted for by the $\sigma_{i}^{b}$s and $\sigma_{i}^{u}$s, which are not shown. Clearly, this uncertainty in the parameters is specific to the individual investigations or the complete data, respectively.

### Parameter correlations and model identifiability

The posterior probabilities of both the HMGU and ICRP model showed strong correlation between the parameters *x*_7_ and *x*_8_ throughout all investigations. The estimated Kendall’s *τ*’s based on the preruns were ${\hat{\tau }}_{\mathrm{HMGU}}=0.8027\pm 0.01$ and ${\hat{\tau }}_{\mathrm{ICRP}}=0.3452\pm 0.02$. This can be explained as follows: At time point *t*=0 the stomach compartment *y*_9_is the only compartment with non-zero Zr concentration. It is exclusively connected to the small intestines *y*_10_ in all models. Therefore, all Zr compounds have to pass through *y*_10_, which further on distributes them to the observed transfer compartment *y*_1_ via *x*_7_ or to the upper large intestines *y*_5_ via *x*_8_. Aberrations in one of the parameters *x*_7_ or *x*_8_ thus have a direct effect on the amount of zirconium predicted for *y*_1_. This affects the according posterior distributions. The same effect is found for the complete data D (compare pairwise scatterplots in Additional file 1 section 4.2). Despite the parameter dependencies, the posterior distributions of the ICRP and HMGU model are identifiable for all 16 investigations, this is, the investigation specific maximum a posteriori estimates are well defined and inferable (cf. Additional file 1 section 4.3).

### Bayesian model comparison

Using the concept of thermodynamic integration we compared the HMGU and the ICRP model based on (i) the concatenated data $D=\{D_{1},\ldots ,D_{16}\}$ and (ii) the individual investigation specific datasets $D_{i}$ (*i*=1,…,16). This results in a total of 17 Bayes factors. We found that all Bayes factors favored the HMGU model; in 14 out of the 17 cases even decisively (cf. Table 1, second column, of this section and section 4 of Additional file 1).

**Table 1:** Bayes factors

**Inv.**	$B_{H,I}^{A}$	$B_{H,I}^{p}$	$B_{H,I}^{u}$
1	7.17·10^1^	7.12·10^1^	1.05
2	1.15·10^2^	2.93·10^2^	3.94·10^3^
3	5.95·10^4^	5.23·10^4^	1.34
4	1.07·10^3^	2.64·10^3^	3.47·10^1^
5	2.19·10^2^	4.73·10^2^	1.34·10^2^
6	4.64·10^3^	3.93·10^3^	2.38·10^3^
7	2.18·10^2^	2.30·10^2^	1.34·10^3^
8	3.75·10^1^	1.28·10^2^	0.22
9	4.62·10^2^	2.32·10^2^	0.18
10	8.62·10^2^	1.16·10^2^	0.20
11	1.17·10^5^	1.81·10^1^	2.94·10^3^
12	1.78·10^2^	5.48	1.14·10^1^
13	7.19·10^2^	1.41·10^1^	4.41
14	3.58·10^1^	7.43	9.77
15	6.29·10^3^	2.17·10^1^	1.60·10^2^
16	6.22·10^2^	1.34·10^1^	1.20·10^4^
ALL	1.20·10^11^	3.43·10^4^	4.73·10^7^

Bayes factors for the HMGU versus the ICRP model ( $B_{H,I}^{A}$) for investigation 1,…,16 and the complete data model (ALL) as well as the according Bayes factors for the plasma ( $B_{H,I}^{p}$) and urine ( $B_{H,I}^{u}$) data. The HMGU model is favored *substantially*, when $B_{H,I}^{\cdot }>3$ and *decisively*, when $B_{H,I}^{\cdot }>100$. Also, $1/B_{H,I}^{\cdot }=B_{I,H}^{\cdot }$.

In order to take a closer look at the contribution of the plasma and urine data to the above results, we computed additional Bayes factors based on the likelihoods $L_{i}^{b}(x^{k},k|D_{i})$ and $L_{i}^{u}(x^{k},k|D_{i})$ individually. Here, *i*=1,…,16,*ALL*and *k*∈{*I*,*H*}, where *I* represents the ICRP and *H* the HMGU model. The time courses already suggested better coverage of plasma data by the HMGU model (Figure 3 above and section 4.4 of Additional file 1); for urine the situation is not that clear. This was confirmed by the Bayes factors (see Additional file 1 section 4 for sampling details): all 17 Bayes factors based on plasma data favored the HMGU model; in ten cases even decisively (Table 1, third column). For the urine data, three investigations slightly favored the ICRP model (Table 1, fourth column). In summary, all decisive Bayes factors are in favor of the HMGU model. While the HMGU model was never decisively rejected, the ICRP model was decisively rejected in the majority of cases. Hence, in depth analysis showed that the HMGU model is superior to the ICRP model with respect to zirconium processing in the human body. This not only holds investigation-specifically, but also based on the complete data. We additionally considered an extension of the HMGU model (see Additional file 1 section 1.2 and 4) which, however, did not show any significant improvements.

### Differences in radioactive ^95^Zr retention in bone predicted by the HMGU and ICRP models

In internal exposure monitoring, biokinetic models were used to predict the organ retention or daily excretion of incorporated radionuclides [44]. With an interval of 120 days the radioactivity of ^95^Zr possibly incorporated by occupational workers was routinely monitored by whole body counters. Depending on the intake route, the radiation dose of bone surfaces or colon was taken as regulatory limit for a decision if an individual is requested for person-specific monitoring [45]. In this monitoring procedure, the biokinetic model structure and parameters are used implicitly in the background. The organ retention function is the solution of the model in each compartment; the organ doses are directly related to the integral of radioactivity of ^95^Zr in source organs over 50 years.

**Figure 4:**
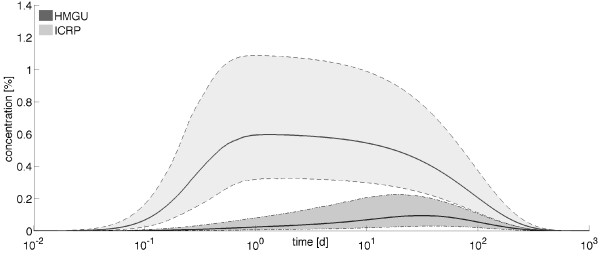
**Zirconium retention in bones.** Median (solid lines) as well as 90% credible intervals (shaded areas) for the retention of ^95^Zr in the bone compartment(s) as predicted by the HMGU and ICRP models, taking into account radioactive decay.

In order to compare the retention of ^95^Zr as predicted by the ICRP and HMGU models, the 90% credible intervals for the time courses in the bone compartments were calculated based on the posterior samples. It is found that there is a significant difference between the two models (Figure 4), where for the ICRP model we added the concentrations in the two bone compartments. The time courses were derived for stable isotopes of Zr and thus had to take the radioactive decay of ^95^Zr with half-life of 64.032 d [46] into account. The decrease of retention in bone using the HMGU model consequently reduces the radiation dose estimate in bone in comparison to the ICRP bone dose which is currently used in monitoring.

### Retrospective dose assessment

Internal doses due to incorporated radionuclides have to be estimated with the help of biokinetic models based on indirect measurements, using for example bioassays for blood or urinary excretion. Normally, bioassay or in vivo data (e.g. radioactivity accumulated in skull or knee detected by a partial body counter) are measured after an accidental intake of radionuclides. Uncertainties of estimated doses are significant and have a large impact on the remediation and thus action costs. In contrast to conventional uncertainty analysis as performed in [7], our Bayesian approach naturally integrates the uncertainties of measured data and parameters simultaneously. This trait of the Bayesian approach is useful as it provides an estimate for the intake and its credible intervals.

For example, if the urinary excretion after accidental exposure is measured, we can compute credible intervals for the initial intake of radionuclide ^95^Zr by exploiting the posterior distribution together with the linearity of the HMGU model. In order to be as general as possible we used the posterior samples based on the complete data. Given a posterior sample ***x***^*H*^, a measurement ${\dot{y}}_{7}^{t}$ in [*μg*/*d*] for the urinary excretion rate of zirconium at time point *t* corresponds to a unique solution $c_{x^{H}}(t)$ of the HMGU ODE system. Due to the linearity of the ODE’s, the initial concentration $c_{x^{H}}(0)$ is by definition zero for all except the stomach compartment *y*_9_. The latter reads $y_{9}(0)={\dot{y}}_{7}^{t}\cdot 100\%/c_{x^{H}}^{9}(t)$ where $c_{x^{H}}^{9}(t)$ denotes the value of $c_{x^{H}}(t)$ in the stomach compartment at time point *t*. Now, assuming that for arbitrary posterior samples ***x***^*H*^ the measurement ${\dot{y}}_{7}^{t}$ is contained in the 90% credible interval of the solution $c_{x^{H}}(t)$ with initial condition *y*_9_(0) as given above, lower and upper bounds for credible regions of the initial amount of zirconium taken in at *t*_0_=0 emerge. These are based on the posterior samples. The estimated extrapolation factors for multiplication with a urine measurement (in [*μ*g/d]) after time *t* (in [*h*]) are contained in Table 2 and yield the initial amount of zirconium contained in the stomach at *t*_0_=0. For example, if an amount of ${\dot{y}}_{7}^{2d}=50\mu$g/d was measured after two days, we find from Table 2 that the 90% credible interval for the ingested amount lies between 0.029g and 0.059g. Since the above calculations are based on non-radioactive Zr isotopes, the results have to be corrected for dose assessment with respect to radioactive decay of the radionuclide in question, i.e. in many cases ^95^Zr.

**Table 2:** Urine predictions for the HMGU model

**Time *t***	**6h**	**12h**	**18h**	**24h**	**30h**
lbf for IC	1233.91	1820.44	2614.48	3369.70	4100.16
mf for IC	1763.73	2225.90	3153.70	4228.19	5340.23
ubf for IC	2512.54	2832.49	3978.27	5650.86	7516.00
Time *t*	36h	42h	48h	54h	60h
lbf for IC	4778.27	5352.64	5800.77	6153.80	6450.74
mf for IC	6364.76	7250.67	7977.31	8557.87	9006.97
ubf for IC	9122.11	10655.01	11878.81	12960.61	13903.07

Shown are the lower bound factor (lbf), median factor (mf), and upper bound factor (ubf) for multiplication with a urine measurement (in [*μ*g/d]) after time *t* (in [h]) on a 60h grid yielding the initial intake concentration (IC) at *t*_0_=0.

## Conclusions

We were the first to evaluate two competing biokinetic ODE models for zirconium processing in the human body after ingestion. These models were the current model recommended by the International Commission on Radiological Protection (ICRP) and a model developed by the Helmholtz Zentrum München (HMGU). The analysis was based on a Bayesian approach, i.e. individual Bayes factors for 16 investigations as well as a Bayes factor based on the concatenated dataset. In order to obtain reliable Monte Carlo sampling results, we combined the numerically stable thermodynamic integration with an efficient copula-based Metropolis-Hastings algorithm. In summary, the HMGU model was unequivocally superior with 14 of 17 Bayes factors being even decisive when compared to the well-established ICRP model. Also, when restricting the data on plasma and urine measurements only, we found that the HMGU model was clearly favored. The HMGU model thus best covers human data.

In contrast to the ICRP model, the HMGU model predicted a delayed accumulation of zirconium in bones which might be experimentally tested in animals in the future. Furthermore, we showed that the HMGU model can be applied for retrospective dose assessment, where the initially ingested amount of zirconium can be reconstructed including credible intervals from *ex post* urine measurements. This provides a simple hands-on tool that facilitates the decision if measures have to be taken in case of accidental exposure. In future applications the superior HMGU model together with its posterior samples can readily be used as the basis for dose estimation in internal dosimetry. The Bayesian framework for the compartmental model analysis developed in the present work is directly applicable to a personalized dose assessment and the uncertainty quantification if a person-specific monitoring is requested. More generally, the presented methodology is suitable for any ODE-based model selection task, such as the modeling of protein signaling, gene regulation, or drug processing [47], nowadays frequently put forward in systems biology [48,49] or pharmacogenetics [50].

## Competing interests

The authors declare that they have no competing interests.

## Authors’ contributions

DS and SH implemented the algorithms, performed the computational simulations, and interpreted the results to an equal degree. DS implemented the copula-based MH algorithm. MBG provided the data. WBL and MBG supplied the models, helped analyze the data, and curated the Bayesian prior information. FJT helped to draft the manuscript and coordinated the theoretical work. DS, SH, WBL, and MBG wrote the manuscript. All authors read and approved the final manuscript.

## Supplementary Material

Additional file 1 **Supplementary information.** Supplementary information, including the detailed ODE systems for both models, the prior information used for the inference and more detailed evaluation of the sampling results, among them additional time course plots for the single investigations and scatterplots for the evaluation of parameter dependencies. Furthermore, we provide an identifiability analysis for all models and a model variant of the HMGU model including its evaluation via Bayes factors. Click here for file
