# Supplementary material for: Bayesian model selection validates a biokinetic model for zirconium processing in humans
Source: BMC Syst Biol. 2012 Aug 5;6:95. doi: 10.1186/1752-0509-6-95 (PMC3529705; doi:10.1186/1752-0509-6-95)
Supplement: Additional file 1 — Supplementary information. Supplementary information, including the detailed ODE systems for both models, the prior information used for the inference and more detailed evaluation of the sampling results, among them additional time course plots for the single investigations and scatterplots for the evaluation of parameter dependencies. Furthermore, we provide an identifiability analysis for all models and a model variant of the HMGU model including its evaluation via Bayes factors. [file 1752-0509-6-95-S1.pdf]

Bayesian model selection validates a biokinetic model for zirconium processing in humans

Daniel Schmidl, Sabine Hug, Wei Bo Li, Matthias B. Greiter & Fabian J. Theis

9th March 2012

Supplementary material

Contents

1 Mathematical model definition **3**
1.1 ICRP model . . . 3
1.2 HMGU model . . . 5
1.3 Solution of the ODE systems . . . 7

2 Prior information **8**
2.1 Location and scale parameters for the lognormal distribution given the estimated median and geometric standard deviation . . . 8
2.2 Mean and standard deviation for the normal distribution given the estimated median and the coefficient of variation . . . 8
2.3 Summary of prior distributions . . . 8

3 Jeffreys' scale of confidence **11**

4 Sampling results **12**
4.1 Regions of highest posterior density . . . 14
4.2 Parameter correlations . . . 14
4.3 Identifiability analysis . . . 18
4.4 Time courses . . . 21

1 Mathematical model definition

1.1 ICRP model

The model for biokinetics of zirconium put forward by the International Commission on Radiological Protection (ICRP) is a compartmental model consisting of eleven compartments $y_1, \ldots, y_{11}$ and 15 reaction rates $x_1, \ldots, x_8, x_{13}, \ldots, x_{19}$ ([ICRP, 1989], [ICRP, 1993]). Zirconium enters the body in the stomach compartment y_9 and diffuses through the system until it reaches either one of the two final compartments urine, y_7, or feces, y_8. The gray compartments y_1 and y_7 are directly related to the data points measured.

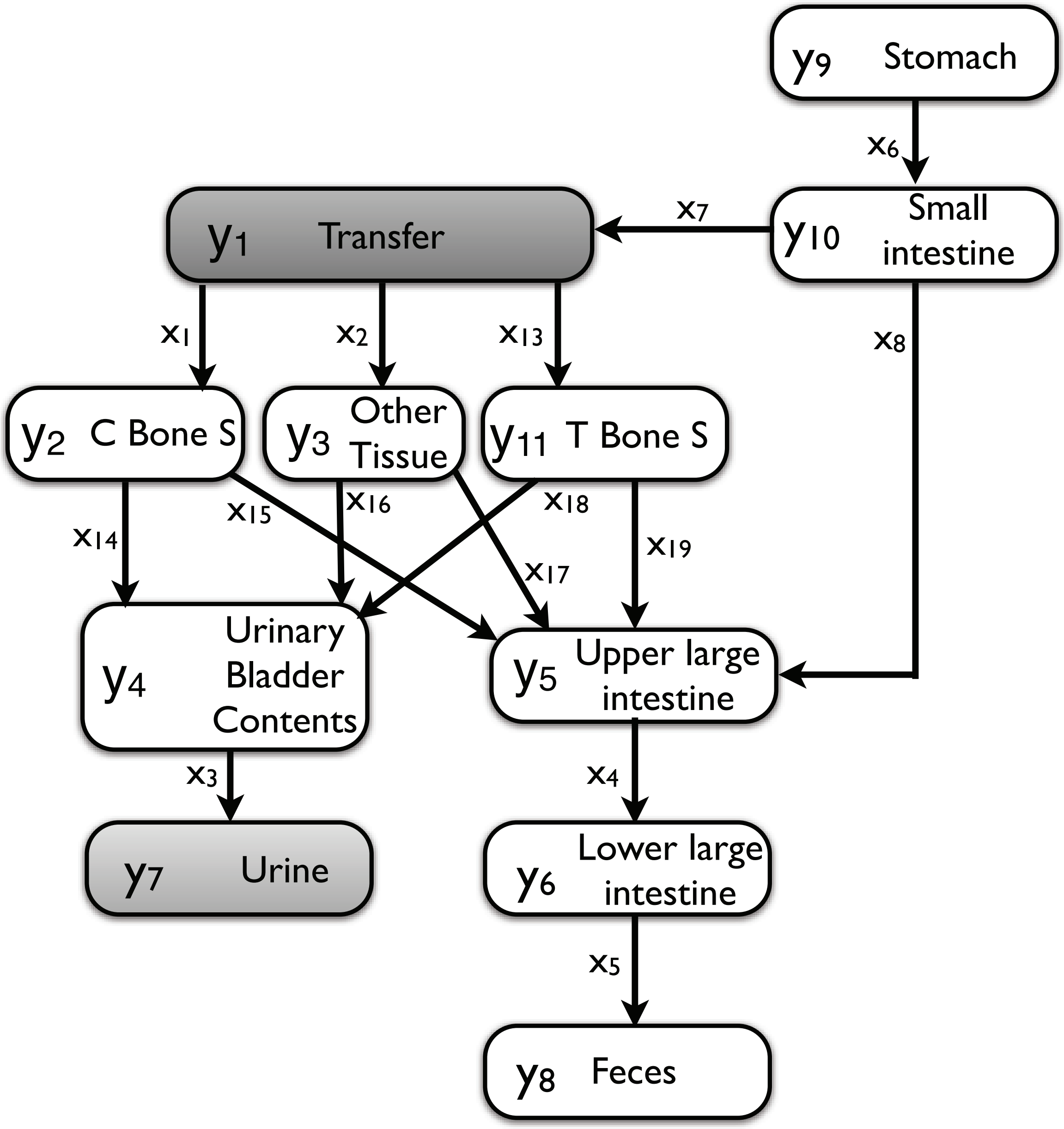

Mathematically, the model is described by the following system of eleven coupled ODEs:

$$
\begin{aligned}
\frac{\mathrm{d}y_1(t)}{\mathrm{d}t} &= (-x_1 - x_2 - x_{13})\, y_1(t) + x_7 y_{10}(t) \\
\frac{\mathrm{d}y_2(t)}{\mathrm{d}t} &= x_1 y_1(t) + (-x_{14} - x_{15})\, y_2(t) \\
\frac{\mathrm{d}y_3(t)}{\mathrm{d}t} &= x_2 y_1(t) + (-x_{16} - x_{17})\, y_3(t) \\
\frac{\mathrm{d}y_4(t)}{\mathrm{d}t} &= x_{14} y_2(t) + x_{16} y_3(t) - x_3 y_4(t) + x_{18} y_{11}(t) \\
\frac{\mathrm{d}y_5(t)}{\mathrm{d}t} &= x_{15} y_2(t) + x_{17} y_3(t) - x_4 y_5(t) + x_8 y_{10}(t) + x_{19} y_{11}(t) \\
\frac{\mathrm{d}y_6(t)}{\mathrm{d}t} &= x_4 y_5(t) - x_5 y_6(t) \\
\frac{\mathrm{d}y_7(t)}{\mathrm{d}t} &= x_3 y_4(t) \\
\frac{\mathrm{d}y_8(t)}{\mathrm{d}t} &= x_5 y_6(t) \\
\frac{\mathrm{d}y_9(t)}{\mathrm{d}t} &= -x_6 y_9(t) \\
\frac{\mathrm{d}y_{10}(t)}{\mathrm{d}t} &= x_6 y_9(t) + (-x_7 - x_8)\, y_{10}(t) \\
\frac{\mathrm{d}y_{11}(t)}{\mathrm{d}t} &= x_{13} y_1(t) + (-x_{18} - x_{19})\, y_{11}(t)
\end{aligned}
$$

with initial conditions

$$
\begin{aligned}
y_1(t=0) &= 0\% \\
y_2(t=0) &= 0\% \\
y_3(t=0) &= 0\% \\
y_4(t=0) &= 0\% \\
y_5(t=0) &= 0\% \\
y_6(t=0) &= 0\% \\
y_7(t=0) &= 0\% \\
y_8(t=0) &= 0\% \\
y_9(t=0) &= 100\% \\
y_{10}(t=0) &= 0\% \\
y_{11}(t=0) &= 0\%.
\end{aligned}
$$

1.2 HMGU model

The model for biokinetics of zirconium put forward by the Helmholtz Zentrum München (HMGU) consists of ten compartments $y_1, \ldots, y_{10}$ and twelve reaction rates $x_1,\ldots,x_{12}$ ([Greiter *et al.*, 2011]). Retrospective dosimetry as done in section 4.6 of the main texts critically depends on the modeling of urinary excretion. Therefore we adapted a scheme used for the processing of strontium ([Li *et al.*, 2008]), i.e. we added an additional urinary path compartment, along with three transfer rates to the HMGU model, hoping for even better urine fits. This *extended HMGU model*, like the ICRP model, contains 15 parameters. The evaluation of the extended HMGU model can be found in section 4.

Explicitly, the extended HMGU model additionally contains the compartment y_{12} and the reaction rates x_{20}, x_{21} and x_{22} (shown in red). In either model zirconium enters the body in the stomach compartment y_9 and diffuses through the system until it reaches either one of the two final compartments urine, y_7, or feces, y_8. Again, the gray compartments y_1 and y_7 are directly related to the data points measured.

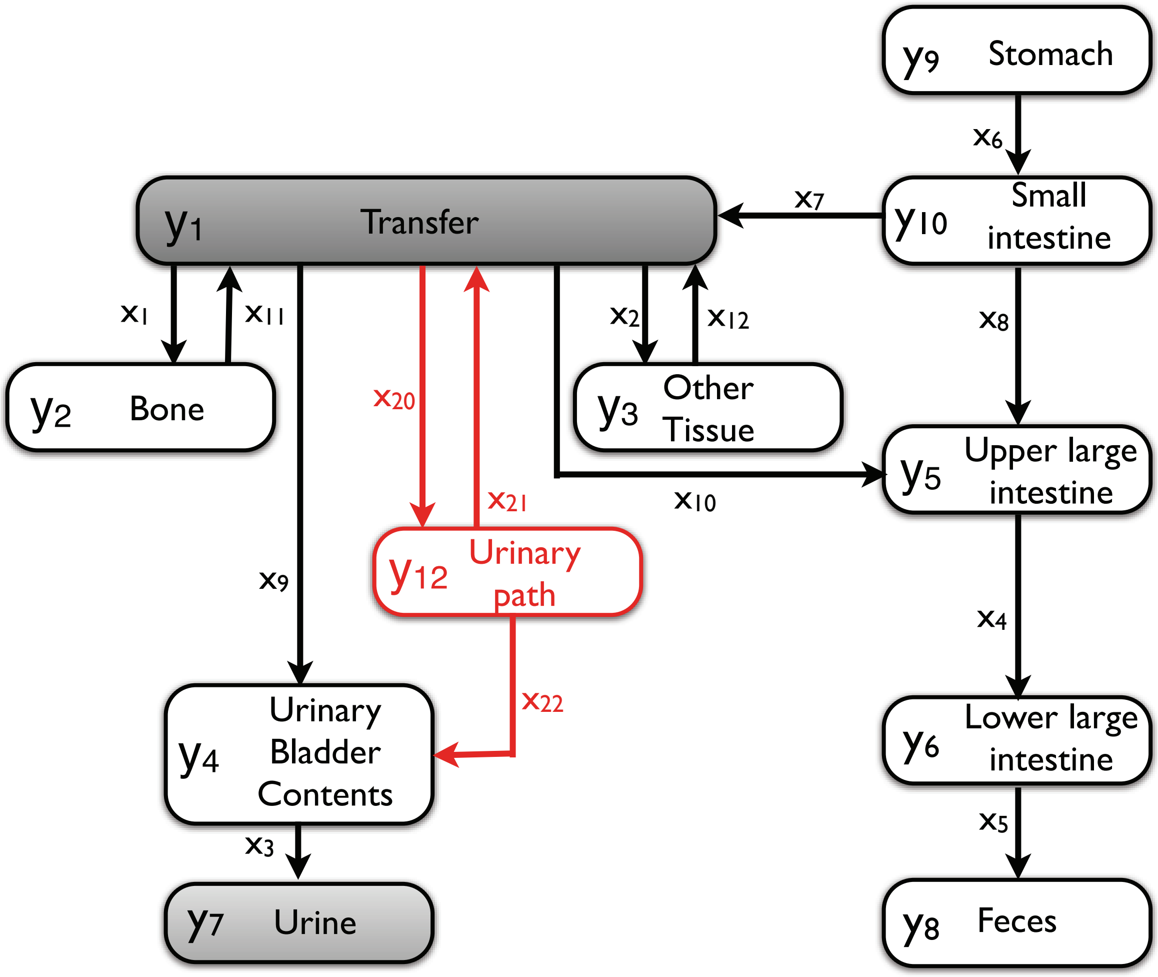

Mathematically, the original HMGU model is described by the following system of coupled ODEs:

$$\begin{aligned}
\frac{\mathrm{d}y_1(t)}{\mathrm{d}t} &= (-x_1 - x_2 - x_9 - x_{10})\, y_1(t) + x_{11} y_2(t) + x_{12} y_3(t) + x_7 y_{10}(t) \\
\frac{\mathrm{d}y_2(t)}{\mathrm{d}t} &= x_1 y_1(t) - x_{11} y_2(t) \\
\frac{\mathrm{d}y_3(t)}{\mathrm{d}t} &= x_2 y_1(t) - x_{12} y_3(t) \\
\frac{\mathrm{d}y_4(t)}{\mathrm{d}t} &= x_9 y_1(t) - x_3 y_4(t) \\
\frac{\mathrm{d}y_5(t)}{\mathrm{d}t} &= x_{10} y_1(t) - x_4 y_5(t) + x_8 y_{10}(t) \\
\frac{\mathrm{d}y_6(t)}{\mathrm{d}t} &= x_4 y_5(t) - x_5 y_6(t) \\
\frac{\mathrm{d}y_7(t)}{\mathrm{d}t} &= x_3 y_4(t) \\
\frac{\mathrm{d}y_8(t)}{\mathrm{d}t} &= x_5 y_6(t) \\
\frac{\mathrm{d}y_9(t)}{\mathrm{d}t} &= -x_6 y_9(t) \\
\frac{\mathrm{d}y_{10}(t)}{\mathrm{d}t} &= x_6 y_9(t) + (-x_7 - x_8)\, y_{10}(t)
\end{aligned}$$

with initial conditions

$$\begin{aligned}
y_1(t=0) &= 0\% \\
y_2(t=0) &= 0\% \\
y_3(t=0) &= 0\% \\
y_4(t=0) &= 0\% \\
y_5(t=0) &= 0\% \\
y_6(t=0) &= 0\% \\
y_7(t=0) &= 0\% \\
y_8(t=0) &= 0\% \\
y_9(t=0) &= 100\% \\
y_{10}(t=0) &= 0\%.
\end{aligned}$$

The extended HMGU model is identical to the original HMGU model except for the following changes (the additional reaction rates and compartment are depicted in red):

$$\begin{aligned}
\frac{\mathrm{d}y_1(t)}{\mathrm{d}t} &= (-x_1 - x_2 - x_9 - x_{10} - x_{20})\, y_1(t) + x_{11} y_2(t) + x_{12} y_3(t) + x_7 y_{10}(t) + x_{21} y_{12}(t) \\
\frac{\mathrm{d}y_4(t)}{\mathrm{d}t} &= x_9 y_1(t) - x_3 y_4(t) + x_{22} y_{12}(t) \\
\frac{\mathrm{d}y_{12}(t)}{\mathrm{d}t} &= x_{20} y_1(t) + (-x_{21} - x_{22})\, y_{12}(t)
\end{aligned}$$

with initial conditions

$$
\begin{aligned}
y_1(t=0) &= 0\% \\
y_2(t=0) &= 0\% \\
y_3(t=0) &= 0\% \\
y_4(t=0) &= 0\% \\
y_5(t=0) &= 0\% \\
y_6(t=0) &= 0\% \\
y_7(t=0) &= 0\% \\
y_8(t=0) &= 0\% \\
y_9(t=0) &= 100\% \\
y_{10}(t=0) &= 0\% \\
y_{12}(t=0) &= 0\%.
\end{aligned}
$$

The initial conditions for compartments $y_1 - y_{10}$ coincide for all models.

1.3 Solution of the ODE systems

For the calculation of the likelihood $\mathcal{L}_i(\boldsymbol{x}^k, k)$, the ODE has to be solved based on the current parameters $\boldsymbol{x}^k$. Since the ODEs at question are of first order, they can be written as

$$\frac{d\mathbf{y}_{\boldsymbol{x}^k}(t)}{dt} = A(\boldsymbol{x}^k) \cdot \mathbf{y}_{\boldsymbol{x}^k}(t), \tag{1}$$

where $\mathbf{y}_{\boldsymbol{x}^k}(t)$ is the vector of all the compartments of model k and the time independent matrix $A(\boldsymbol{x}^k)$ represents all the interactions between these compartments, depending on the transfer rate values $\boldsymbol{x}^k$. The analytical solution is then given by

$$\mathbf{y}_{\boldsymbol{x}^k}(t) = e^{A(\boldsymbol{x}^k)t} \cdot \mathbf{y}_{\boldsymbol{x}^k}(t=0). \tag{2}$$

The matrix exponential $e^{A(\boldsymbol{x}^k)t}$ was computed by decomposing

$$A(\boldsymbol{x}^k) = U(\boldsymbol{x}^k)\ \mathrm{diag}\left(d_1(\boldsymbol{x}^k), d_2(\boldsymbol{x}^k), \ldots, d_V(\boldsymbol{x}^k)\right)\ U(\boldsymbol{x}^k)^{-1} \tag{3}$$

such that

$$e^{A(\boldsymbol{x}^k)t} = U(\boldsymbol{x}^k) \begin{pmatrix} e^{d_1(\boldsymbol{x}^k)t} & \cdots & \cdots & 0 \\ \vdots & e^{d_2(\boldsymbol{x}^k)t} & & \vdots \\ \vdots & & \ddots & \vdots \\ 0 & \cdots & \cdots & e^{d_V(\boldsymbol{x}^k)t} \end{pmatrix} U(\boldsymbol{x}^k)^{-1} \tag{4}$$

for the eigenvalues $d_1(\boldsymbol{x}^k), d_2(\boldsymbol{x}^k), \ldots, d_V(\boldsymbol{x}^k)$ of $A(\boldsymbol{x}^k)$. For the HMGU model V=10, for the extended HMGU model and the ICRP model V=11. In our case, the eigenvalues and matrices $U(\boldsymbol{x}^k), U(\boldsymbol{x}^k)^{-1}$ were numerically approximated by MATLAB's `eig` function.

By using the `eig` function, we of course introduce numerical error to our solution, however we verified that it is of the same order as the error made by the `ode45` function, yet calculations are much faster. Also, if the analytical solution is available, it is clear that it should be preferred to a purely numerical solution.

2 Prior information

The prior distributions of the HMGU and ICRP model were computed in [Li *et al.*, 2011a]. Since only estimated geometric standard deviations or coefficients of variation as well as estimated medians were provided, we need to infer the location (μ) and scale (σ) parameters for all lognormal distributions $\mathcal{LN}(\mu, \sigma)$. Furthermore, the means μ and standard deviations σ for all normal distributions $\mathcal{N}(\mu, \sigma)$ need to be computed. The formulas are derived in the following.

2.1 Location and scale parameters for the lognormal distribution given the estimated median and geometric standard deviation

Suppose we are given the estimated $\hat{m}$ for the median m of a univariate lognormal distribution $\mathcal{LN}(\mu, \sigma)$.
According to [Johnson, 1994], $m = \exp(\mu)$ and therefore

$$\mu = \log(m) \approx \log(\hat{m}).$$

Furthermore, the geometric standard deviation (GSD) is provided for all lognormally distributed parameters and is either $GSD = 2$ or $GSD = 3$. Since for a lognormal distribution, we also have $GSD = \exp(\sigma)$, this naturally yields

$$\sigma = \ln(2)$$

or

$$\sigma = \ln(3)$$

for the scale parameter.

2.2 Mean and standard deviation for the normal distribution given the estimated median and the coefficient of variation

Since for the normal distribution $\mathcal{N}(\mu, \sigma)$ the mean and median coincide, we do not further distinguish between the two of them and simply denote their estimates by $\hat{\mu}$. Clearly, we have

$$\mu \approx \hat{\mu}.$$

Also, for the normally distributed parameters, we are given a coefficient of variation of $c_V = 0.3$. Since $c_V = \frac{\sigma}{\mu}$, we obtain

$$\sigma = 0.3\mu.$$

2.3 Summary of prior distributions

The following tables provide an overview of the prior distributions and distribution parameters used for parameter inference in the HMGU and ICRP model. The values for μ and σ are computed as described in sections 2.1 and 2.2.

The prior distribution for the ICRP model are directly derived from the recommendations of the ICRP and thus well-established over the years. The priors for the HMGU model are also in part derived from these recommendations, plus information gained from additional experiments based on injected Zirconium doses ([Li *et al.*, 2011a])

A keen reader might notice the difference in orders of magnitude for the σs of the HMGU model and the ICRP model. However, these stem mostly from the fact, that the HMGU model has lognormally distributed priors where the ICRP model has normally distributed ones, both models assume a coverage factor of 3 to represent 99.7% confidence interval (ISO GUM, 1995) for normal and lognormal distributions.

Table 1: Overview of priors. The abbreviation are: $\mathcal{LN}(\mu, \sigma)$ = lognormal distribution with location parameter μ and scale parameter σ, $\mathcal{T}(a, b, c)$ = triangular distribution with lower limit a, upper limit b, and mode c.TC= Transfer compartment; CBS = Cortical Bone Surface; Other = Other Tissues; UBC = Urine Bladder Contents; UpLi = Upper Large Intestine; LoLI = Lower Large Intestine; SI = Small Intestine; TBS= Trabecular Bone Surface.

HMGU model

Param.	Compartments	Median (d^{-1})	99.7% confidence interval	distribution	μ	σ	a	c	b
x_1	TC → Bone	0.10	[0.013, 0.8]	$\mathcal{LN}(\mu, \sigma)$	-2.3026	0.6931			
x_2	TC → Other	1.35	[0.17, 10.8]	$\mathcal{LN}(\mu, \sigma)$	0.3001	0.6931			
x_3	UBC → Urine	12.0		$\mathcal{T}(a, b, c)$			6.0	8.0	24.0
x_4	UpLI → LoLI	1.8		$\mathcal{T}(a, b, c)$			0.9	1.2	3.6
x_5	LoLI → Feces	1.0		$\mathcal{T}(a, b, c)$			0.3	1.0	1.7
x_6	Stomach → SI	24.0		$\mathcal{T}(a, b, c)$			12.0	16.0	48.0
x_7	SI → TC	0.03	$[1.11 \cdot 10^{-3}, 0.81]$	$\mathcal{LN}(\mu, \sigma)$	-3.5066	1.0986			
x_8	SI → UpLI	17.21	[0.64, 464.67]	$\mathcal{LN}(\mu, \sigma)$	2.8455	1.0986			
x_9	TC → UBC	0.031	[0.0011, 0.8370]	$\mathcal{LN}(\mu, \sigma)$	-3.4738	1.0986			
x_{10}	TC → UpLI	0.0062	[0.0002, 0.1674]	$\mathcal{LN}(\mu, \sigma)$	-5.0832	1.0986			
x_{11}	Bone → TC	$6.93 \cdot 10^{-5}$	$[8.66 \cdot 10^{-6}, 5.55 \cdot 10^{-4}]$	$\mathcal{LN}(\mu, \sigma)$	-9.5769	0.6931			
x_{12}	Other → TC	0.53	[0.066, 4.24]	$\mathcal{LN}(\mu, \sigma)$	-0.6349	0.6931			

extended HMGU model

Param.	Compartments	Median (d^{-1})	99.7% confidence interval	distribution	μ	σ	a	c	b
x_1	TC → Bone	0.10	[0.013, 0.8]	$\mathcal{LN}(\mu, \sigma)$	-2.3026	0.6931			
x_2	TC → Other	1.35	[0.17, 10.8]	$\mathcal{LN}(\mu, \sigma)$	0.3001	0.6931			
x_3	UBC → Urine	12.0		$\mathcal{T}(a, b, c)$			6.0	8.0	24.0
x_4	UpLI → LoLI	1.8		$\mathcal{T}(a, b, c)$			0.9	1.2	3.6
x_5	LoLI → Feces	1.0		$\mathcal{T}(a, b, c)$			0.3	1.0	1.7
x_6	Stomach → SI	24.0		$\mathcal{T}(a, b, c)$			12.0	16.0	48.0
x_7	SI → TC	0.03	$[1.11 \cdot 10^{-3}, 0.81]$	$\mathcal{LN}(\mu, \sigma)$	-3.5066	1.0986			
x_8	SI → UpLI	17.21	[0.64, 464.67]	$\mathcal{LN}(\mu, \sigma)$	2.8455	1.0986			
x_9	TC → UBC	0.0078	[0.0003, 0.2106]	$\mathcal{LN}(\mu, \sigma)$	-4.8536	1.0986			
x_{10}	TC → UpLI	0.0062	[0.0002, 0.1674]	$\mathcal{LN}(\mu, \sigma)$	-5.0832	1.0986			
x_{11}	Bone → TC	$6.93 \cdot 10^{-5}$	$[8.66 \cdot 10^{-6}, 5.55 \cdot 10^{-4}]$	$\mathcal{LN}(\mu, \sigma)$	-9.5769	0.6931			
x_{12}	Other → TC	0.53	[0.066, 4.24]	$\mathcal{LN}(\mu, \sigma)$	-0.6349	0.6931			
x_{20}	TC → UP	0.02		$\mathcal{LN}(\mu, \sigma)$	-3.6535	1.0986			
x_{21}	UP → TC	0.14		$\mathcal{LN}(\mu, \sigma)$	-1.9661	0.6931			
x_{22}	UP → UBC	1.25		$\mathcal{LN}(\mu, \sigma)$	0.2231	0.6931			

ICRP model

Par.	Compartments	Median (d^{-1})	99.7% confidence interval	distribution	μ	σ	a	c	b
x_1	TC → CBS	0.69	[0.086, 5.52]	$\mathcal{LN}(\mu, \sigma)$	-0.3711	0.6931			
x_2	TC → Other	1.39	[0.174, 11.12]	$\mathcal{LN}(\mu, \sigma)$	0.3293	0.6931			
x_3	UBC → Urine	12		$\mathcal{T}(a, b, c)$			6	8	24
x_4	UpLI → LoLI	1.8		$\mathcal{T}(a, b, c)$			0.9	1.2	3.6
x_5	LoLI → Feces	1		$\mathcal{T}(a, b, c)$			0.3	1	1.7
x_6	Stomach → SI	24		$\mathcal{T}(a, b, c)$			12	16	48
x_7	SI → TC	0.06	[0.0075, 0.48]	$\mathcal{LN}(\mu, \sigma)$	-2.8134	0.6931			
x_8	SI → UpLI	6		$\mathcal{T}(a, b, c)$			3	4	12
x_{13}	TC → TBS	0.69	[0.086, 5.52]	$\mathcal{LN}(\mu, \sigma)$	-0.3711	0.6931			
x_{14}	CBS → UBC	$5.78 \cdot 10^{-5}$	$[5.78 \cdot 10^{-6}, 1.1 \cdot 10^{-4}]$	$\mathcal{N}(\mu, \sigma)$	$5.78 \cdot 10^{-5}$	$1.73 \cdot 10^{-5}$			
x_{15}	CBS → UpLI	$1.16 \cdot 10^{-5}$	$[1.16 \cdot 10^{-6}, 2.2 \cdot 10^{-5}]$	$\mathcal{N}(\mu, \sigma)$	$1.16 \cdot 10^{-5}$	$3.48 \cdot 10^{-6}$			
x_{16}	Other → UBC	0.083	[0.0083, 0.158]	$\mathcal{N}(\mu, \sigma)$	0.083	0.025			
x_{17}	Other → UpLI	0.0165	[0.00165, 0.0314]	$\mathcal{N}(\mu, \sigma)$	0.0165	0.00495			
x_{18}	TBS → UBC	$5.78 \cdot 10^{-5}$	$[5.78 \cdot 10^{-6}, 1.1 \cdot 10^{-4}]$	$\mathcal{N}(\mu, \sigma)$	$5.78 \cdot 10^{-5}$	$1.73 \cdot 10^{-5}$			
x_{19}	TBS → UpLI	$1.16 \cdot 10^{-5}$	$[1.16 \cdot 10^{-6}, 2.2 \cdot 10^{-5}]$	$\mathcal{N}(\mu, \sigma)$	$1.16 \cdot 10^{-5}$	$3.48 \cdot 10^{-6}$			

3 Jeffreys' scale of confidence

In [Jeffreys, 1998], Jeffreys gives an interpretation for the Bayes factor $B_{k,k'}$, where model k is favored over model k' according to Table 2. In case of $B_{k,k'} < 1$, the statements in Table 2 hold for $B_{k',k} = 1/B_{k,k'}$.

Table 2: Jeffreys' scale of evidence.

Bayes factor (BF)	Strength of evidence
$1 <$ BF < 3	Barely worth mentioning
$3 <$ BF < 10	Substantial
$10 <$ BF < 30	Strong
$30 <$ BF < 100	Very strong
BF > 100	Decisive

4 Sampling results

The following tables contain all details on the sampling outcomes for the individual Bayes factors. Table 3 shows the outcome for the combined plasma and urine data, table 4 the outcome when only using the plasma data and table 5 the outcome when using only the urine data. For every Bayes factor

$$B^i_{k,k'} = \frac{p(\mathcal{D}_i|k)}{p(\mathcal{D}_i|k')} \tag{5}$$

the numerator was computed on 30 temperature levels each based on 30,000 MCMC proposals (compare Section 3 in main text). The same holds true for the denominator. The tables column-wise contain (i) the investigation number i or ALL for the complete data model; (ii) the Bayes factor $B^i_{H,eH}$ for the HMGU model against the extended HMGU model, red color as in favor of the HMGU model, green in favor of the extended HMGU model; (iii) the Bayes factor $B^i_{H,I}$ for the HMGU model against the ICRP model, green color indicates a BF in favor of the HMGU model, red color one in favor of the ICRP model; (iv) the Bayes factor $B^i_{eH,I}$ for the extended HMGU model against the ICRP model, green color as in favor of the extended HMGU model, red in favor of the ICRP model; (v),(vi),(vii) the minimal, median and maximal acceptance rates (AR) of the MCMC sampling procedure over all 30 temperatures . The last three columns (viii),(ix),(x) finally contain the minimal, median and maximal effective sampling size (ESS) for all three models.

The extended HMGU model is favored throughout compared to the ICRP model. Comparing the extended HMGU model with the original HMGU model we however found that ten investigations, and thus the majority, had a Bayes factor in favor of the original model. One of them was even decisive. For urinary data, the Bayes factor of the original model versus the extended model is in favor of the original model for all but three investigations. Since Bayes factors take into account the entire posterior distributions, they naturally correct for overfitting issues. Overall, we conclude that the original HMGU model covers the data best compared to the ICRP and extended HMGU models.

Table 3: Sampling performance of the copula based independence sampler on all three models

Inv.	$B^i_{H,eH}$	$B_{H,I}$	$B^i_{eH,I}$	HMGU AR [min, med, max]	eHMGU AR [min, med, max]	ICRP AR [min, med, max]	HMGU samples [min, med, max]	eHMGU samples [min, med, max]	ICRP samples [min, med, max]
ALL	17.6047	$1.2010 \cdot 10^{11}$	$6.8222 \cdot 10^{9}$	[0.54, 0.57, 0.64]	[0.15, 0.51, 0.58]	[0.27, 0.57, 0.60]	[1500, 4643, 10000]	[625, 4018, 10000]	[639, 4643, 10000]
1	1.1582	71.7283	61.9335	[0.56, 0.65, 0.70]	[0.42, 0.61, 0.66]	[0.55, 0.63, 0.67]	[1000, 6000, 15000]	[261, 7500, 10000]	[2308, 6000, 10000]
2	1.1557	114.6109	99.1744	[0.59, 0.65, 0.69]	[0.51, 0.54, 0.56]	[0.54, 0.56, 0.58]	[2000, 7500, 15000]	[289, 3000, 7500]	[1035, 5000, 10000]
3	0.9283	59532.2127	64131.0817	[0.42, 0.61, 0.66]	[0.40, 0.57, 0.65]	[0.47, 0.60, 0.66]	[181, 5500, 10000]	[968, 5500, 15000]	[1667, 6000, 15000]
4	0.7688	1065.3125	1385.6330	[0.63, 0.66, 0.68]	[0.52, 0.59, 0.63]	[0.55, 0.62, 0.64]	[2500, 7500, 15000]	[2308, 6750, 15000]	[811, 6000, 15000]
5	1.4955	219.0939	146.4998	[0.60, 0.66, 0.69]	[0.58, 0.61, 0.64]	[0.56, 0.62, 0.65]	[3750, 7500, 15000]	[1765, 5500, 10000]	[1579, 6000, 15000]
6	1.7465	4642.8755	2658.4107	[0.61, 0.63, 0.68]	[0.58, 0.61, 0.63]	[0.57, 0.62, 0.66]	[1072, 6000, 15000]	[1035, 5000, 15000]	[1765, 6000, 10000]
7	0.1836	218.0765	1187.4842	[0.62, 0.66, 0.71]	[0.31, 0.56, 0.66]	[0.60, 0.64, 0.67]	[1667, 7500, 15000]	[750, 4286, 10000]	[4286, 7500, 15000]
8	0.8293	37.5182	45.2386	[0.47, 0.61, 0.71]	[0.48, 0.59, 0.67]	[0.59, 0.64, 0.69]	[2308, 7500, 15000]	[2728, 5500, 15000]	[3334, 7500, 15000]
9	0.1030	462.3241	4488.1242	[0.48, 0.57, 0.71]	[0.30, 0.49, 0.67]	[0.41, 0.63, 0.67]	[770, 6000, 15000]	[790, 5000, 15000]	[698, 5500, 15000]
10	0.2548	861.7574	3381.4779	[0.43, 0.60, 0.71]	[0.16, 0.41, 0.67]	[0.44, 0.64, 0.68]	[2000, 7500, 15000]	[250, 2864, 10000]	[126, 5000, 10000]
11	38.7192	117250.4521	3028.2253	[0.38, 0.49, 0.63]	[0.08, 0.43, 0.57]	[0.49, 0.57, 0.59]	[1072, 4286, 15000]	[165, 2322, 7500]	[698, 4286, 7500]
12	1.6931	177.9964	105.1323	[0.26, 0.61, 0.72]	[0.28, 0.60, 0.67]	[0.46, 0.62, 0.68]	[313, 5000, 10000]	[715, 4643, 10000]	[2308, 6000, 15000]
13	12.6123	718.7546	56.9884	[0.10, 0.44, 0.70]	[0.49, 0.55, 0.58]	[0.53, 0.58, 0.60]	[169, 4018, 15000]	[304, 3750, 10000]	[2308, 4643, 10000]
14	2.9665	35.8079	12.0709	[0.09, 0.41, 0.69]	[0.27, 0.61, 0.67]	[0.56, 0.64, 0.69]	[345, 3000, 15000]	[1250, 5000, 15000]	[1500, 7500, 15000]
15	1.0935	6287.6538	5749.8051	[0.22, 0.53, 0.70]	[0.12, 0.44, 0.67]	[0.46, 0.64, 0.68]	[121, 5500, 15000]	[202, 3750, 15000]	[1000, 5000, 15000]
16	274.8282	622.4126	2.2647	[0.23, 0.56, 0.64]	[0.17, 0.54, 0.59]	[0.51, 0.58, 0.59]	[417, 3000, 10000]	[276, 2738, 10000]	[1765, 5000, 10000]

Table 4: Sampling performance of the copula based independence sampler on all three models - plasma only

Inv.	$B^i_{H,eH}$	$B_{H,I}$	$B^i_{eH,I}$	HMGU AR [min, med, max]	eHMGU AR [min, med, max]	ICRP AR [min, med, max]	HMGU samples [min, med, max]	eHMGU samples [min, med, max]	ICRP samples [min, med, max]
ALL	0.9893	34283.1711	34654.8476	[0.56, 0.61, 0.64]	[0.50, 0.55, 0.58]	[0.41, 0.57, 0.60]	[1000, 6750, 10000]	[122, 4018, 7500]	[1875, 5000, 10000]
1	0.9758	71.1549	72.9191	[0.53, 0.65, 0.69]	[0.43, 0.54, 0.57]	[0.56, 0.62, 0.66]	[834, 6000, 30000]	[834, 3334, 7500]	[455, 6000, 15000]
2	1.0307	293.4270	284.6958	[0.58, 0.64, 0.67]	[0.54, 0.61, 0.65]	[0.59, 0.62, 0.66]	[338, 7500, 15000]	[2308, 7500, 15000]	[546, 7500, 10000]
3	1.3681	52297.4330	38225.0934	[0.45, 0.62, 0.66]	[0.18, 0.39, 0.53]	[0.55, 0.61, 0.65]	[1200, 6000, 15000]	[417, 2250, 7500]	[1765, 6000, 15000]
4	1.6415	2639.9965	1608.2949	[0.56, 0.60, 0.63]	[0.37, 0.51, 0.55]	[0.50, 0.56, 0.57]	[2308, 6000, 15000]	[133, 3000, 6000]	[968, 3334, 10000]
5	1.1345	473.1182	417.0417	[0.59, 0.65, 0.69]	[0.58, 0.62, 0.65]	[0.59, 0.63, 0.68]	[3334, 8750, 15000]	[2308, 6750, 15000]	[1429, 7500, 15000]
6	1.0266	3926.9639	3825.0673	[0.62, 0.65, 0.70]	[0.59, 0.62, 0.65]	[0.48, 0.62, 0.66]	[577, 7500, 10000]	[3000, 7500, 15000]	[698, 6000, 10000]
7	0.7113	229.9698	323.3228	[0.55, 0.64, 0.72]	[0.55, 0.61, 0.67]	[0.59, 0.64, 0.68]	[968, 6000, 15000]	[1429, 6750, 15000]	[2143, 6750, 15000]
8	2.5451	127.7723	50.2025	[0.38, 0.57, 0.72]	[0.49, 0.62, 0.68]	[0.56, 0.64, 0.69]	[667, 6750, 15000]	[1667, 7500, 15000]	[2308, 7500, 15000]
9	0.1407	231.8086	1647.7474	[0.50, 0.57, 0.65]	[0.31, 0.49, 0.68]	[0.58, 0.65, 0.69]	[653, 4286, 15000]	[1250, 5000, 15000]	[3334, 7500, 15000]
10	0.4999	115.6091	231.2512	[0.53, 0.61, 0.65]	[0.45, 0.49, 0.59]	[0.52, 0.58, 0.60]	[215, 4643, 15000]	[385, 2614, 7500]	[1667, 6000, 10000]
11	0.7661	18.0543	23.5678	[0.56, 0.65, 0.71]	[0.58, 0.64, 0.67]	[0.59, 0.65, 0.69]	[1000, 5500, 15000]	[1579, 7500, 15000]	[3750, 6750, 15000]
12	0.6516	5.4764	8.4041	[0.55, 0.61, 0.64]	[0.47, 0.54, 0.58]	[0.56, 0.58, 0.60]	[750, 6000, 15000]	[698, 3542, 10000]	[2728, 5000, 15000]
13	2.4175	14.1274	5.8438	[0.50, 0.67, 0.71]	[0.55, 0.63, 0.68]	[0.60, 0.65, 0.67]	[1154, 7500, 15000]	[667, 6000, 15000]	[4286, 7500, 15000]
14	1.1759	7.4250	6.3141	[0.59, 0.67, 0.72]	[0.34, 0.62, 0.67]	[0.62, 0.65, 0.69]	[2500, 7500, 15000]	[509, 7500, 15000]	[2728, 8750, 15000]
15	2.3948	21.6865	9.0555	[0.56, 0.61, 0.65]	[0.50, 0.55, 0.58]	[0.55, 0.58, 0.59]	[750, 7500, 15000]	[1500, 4643, 10000]	[2000, 5000, 10000]
16	0.7980	13.4114	16.8069	[0.56, 0.66, 0.70]	[0.42, 0.59, 0.67]	[0.59, 0.66, 0.68]	[625, 7500, 30000]	[255, 5500, 10000]	[3334, 7500, 15000]

Table 5: Sampling performance of the copula based independence sampler on all three models - urine only

Inv.	$B^i_{H,eH}$	$B_{H,I}$	$B^i_{eH,I}$	HMGU AR [min, med, max]	eHMGU AR [min, med, max]	ICRP AR [min, med, max]	HMGU samples [min, med, max]	eHMGU samples [min, med, max]	ICRP samples [min, med, max]
ALL	13.8294	47303749.2905	3420524.7980	[0.42, 0.58, 0.65]	[0.14, 0.53, 0.58]	[0.37, 0.57, 0.60]	[35, 4286, 15000]	[192, 2614, 10000]	[600, 4286, 10000]
1	1.1019	1.0460	0.9493	[0.66, 0.70, 0.73]	[0.58, 0.66, 0.68]	[0.64, 0.66, 0.68]	[5000, 10000, 15000]	[3334, 7500, 15000]	[3750, 7500, 15000]
2	34.8195	3940.3951	113.1665	[0.35, 0.54, 0.64]	[0.12, 0.53, 0.58]	[0.54, 0.57, 0.60]	[770, 4643, 10000]	[264, 2728, 10000]	[2500, 5000, 7500]
3	1.3446	1.3352	0.9930	[0.59, 0.70, 0.73]	[0.61, 0.66, 0.70]	[0.60, 0.67, 0.70]	[2143, 8750, 15000]	[2143, 7500, 15000]	[4286, 7500, 15000]
4	14.7689	34.7362	2.3520	[0.46, 0.65, 0.72]	[0.16, 0.62, 0.67]	[0.59, 0.65, 0.69]	[380, 6000, 15000]	[219, 5500, 15000]	[2143, 7500, 15000]
5	55.4583	133.8984	2.4144	[0.43, 0.59, 0.64]	[0.47, 0.56, 0.58]	[0.55, 0.58, 0.60]	[244, 4018, 15000]	[1429, 4286, 7500]	[2308, 5000, 10000]
6	1030.8867	2384.2435	2.3128	[0.13, 0.48, 0.63]	[0.48, 0.56, 0.59]	[0.58, 0.61, 0.62]	[257, 3000, 15000]	[1072, 3334, 7500]	[1667, 4286, 7500]
7	793.4081	1335.8332	1.6837	[0.13, 0.50, 0.63]	[0.50, 0.56, 0.58]	[0.58, 0.61, 0.62]	[136, 3167, 10000]	[1579, 4286, 7500]	[2500, 4286, 10000]
8	0.5550	0.2221	0.4001	[0.57, 0.69, 0.72]	[0.60, 0.66, 0.68]	[0.58, 0.66, 0.68]	[3750, 10000, 15000]	[2728, 7500, 15000]	[3334, 7500, 15000]
9	0.2851	0.1753	0.6149	[0.33, 0.62, 0.70]	[0.24, 0.56, 0.68]	[0.46, 0.63, 0.68]	[235, 4286, 10000]	[625, 6000, 15000]	[770, 7500, 15000]
10	0.7125	0.1992	0.2796	[0.48, 0.68, 0.71]	[0.44, 0.64, 0.68]	[0.58, 0.64, 0.69]	[1154, 10000, 30000]	[2308, 6000, 15000]	[2000, 6750, 15000]
11	13.9183	2936.7417	210.9992	[0.33, 0.48, 0.63]	[0.14, 0.48, 0.59]	[0.52, 0.57, 0.60]	[273, 3542, 15000]	[327, 1938, 7500]	[2143, 5000, 7500]
12	2.8094	11.4359	4.0706	[0.51, 0.59, 0.64]	[0.31, 0.54, 0.58]	[0.50, 0.57, 0.60]	[546, 5500, 10000]	[338, 3750, 10000]	[1072, 5000, 7500]
13	4.6281	4.4105	0.9530	[0.48, 0.64, 0.71]	[0.58, 0.66, 0.69]	[0.59, 0.65, 0.69]	[1000, 6750, 15000]	[3334, 7500, 15000]	[2728, 7500, 15000]
14	2.5476	9.7741	3.8366	[0.43, 0.54, 0.63]	[0.39, 0.55, 0.58]	[0.53, 0.57, 0.60]	[968, 5000, 15000]	[556, 3167, 10000]	[1875, 5000, 10000]
15	2.5774	160.0045	62.0801	[0.40, 0.61, 0.71]	[0.19, 0.53, 0.66]	[0.52, 0.63, 0.68]	[320, 5000, 15000]	[192, 3750, 15000]	[1875, 6000, 15000]
16	2.4061	12003.8714	4989.0320	[0.30, 0.50, 0.63]	[0.06, 0.13, 0.57]	[0.54, 0.61, 0.62]	[366, 3334, 7500]	[53, 252, 6000]	[1500, 3750, 10000]

4.1 Regions of highest posterior density

We derived the 95% credible intervals, i.e. the regions of highest posterior density from the posterior samples of the complete data run, see table 6. Furthermore, we also give the maximum a posteriori (MAP) estimate as the sample with the highest posterior value. From a comparison with table 1, one can see that some parameters are slightly shifted. Since these parameter values are derived from the concatenated data, they are valid for all investigations and thus represent the parameters of choice for an average subject, where no individual information or measurements are available.

Table 6: credible intervals for best parameters - from the posterior samples of the complete data

Param.	x_1	x_2	x_3	x_4
95% CI	[0.03,0.42]	[0.63,2.99]	[7.14,20.91]	[1.03,3.18]
MAP	0.08	1.48	9.54	1.28
Param.	x_5	x_6	x_7	x_8
95% CI	[0.47,1.55]	[17.57,45.15]	[0.10,0.61]	[19.58,134.48]
MAP	1.03	37.43	0.19	41.86
Param.	x_9	x_{10}	x_{11}	x_{12}
95% CI	[0.12,0.28]	[$6.75 \cdot 10^{-4}$,0.06]	[$1.86 \cdot 10^{-5}$,$2.57 \cdot 10^{-4}$]	[0.14,0.82]
MAP	0.20	0.0028	$3.57 \cdot 10^{-5}$	0.27

4.2 Parameter correlations

The following three figures 1, 2 and 3 show the pairwise scatterplots in the lower left triangle, the histogramm of the samples for the parameters on the diagonal and the pairwise Kendall's τs in the upper right triangle. The figures are based on the prerun samples for the complete data model. Note that most correlations are weak at best, except the one between x_7 and x_8 in all three models. The scatterplots for the individual investigations look very similar and are thus omitted.

Figure 1: Scatterplot and Kendall's τs for the HMGU model based on the complete data runs

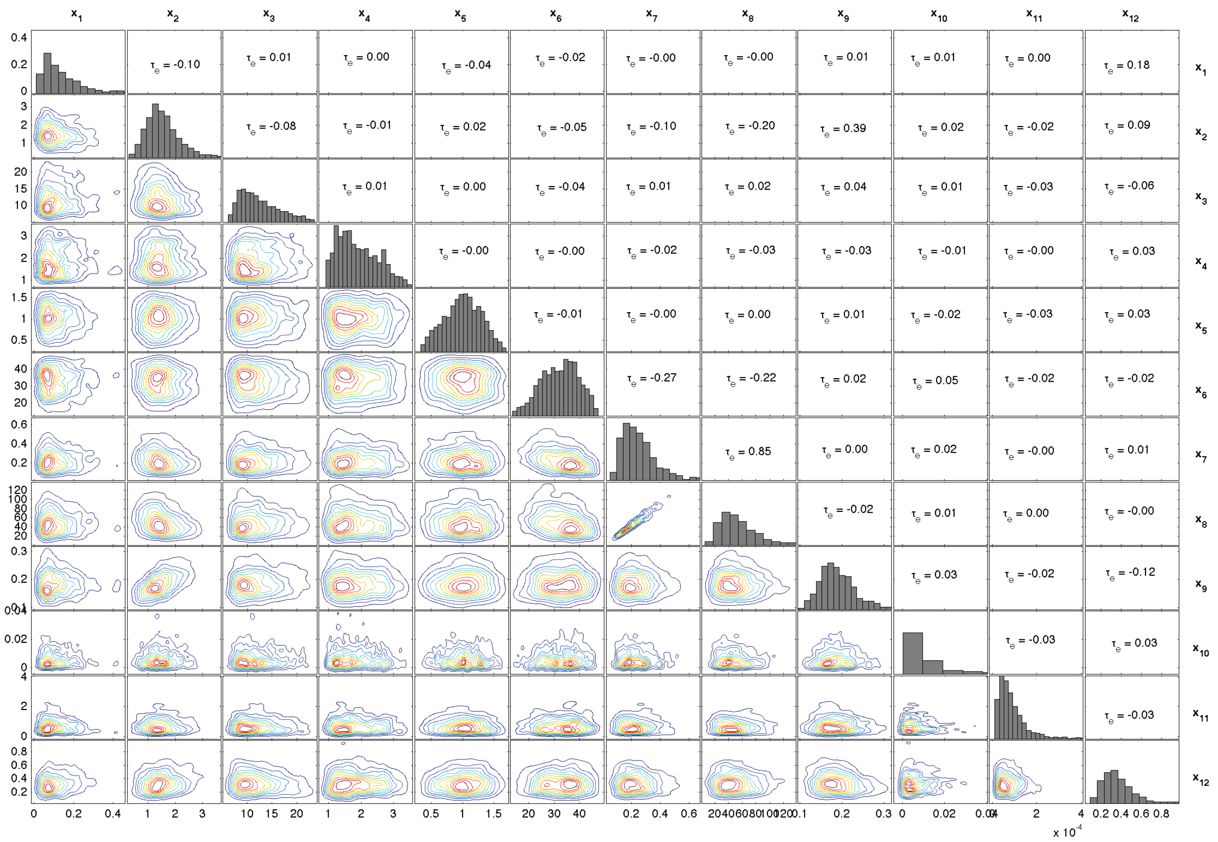

Figure 2: Scatterplot and Kendall's τs for the extended HMGU model based on the complete data runs

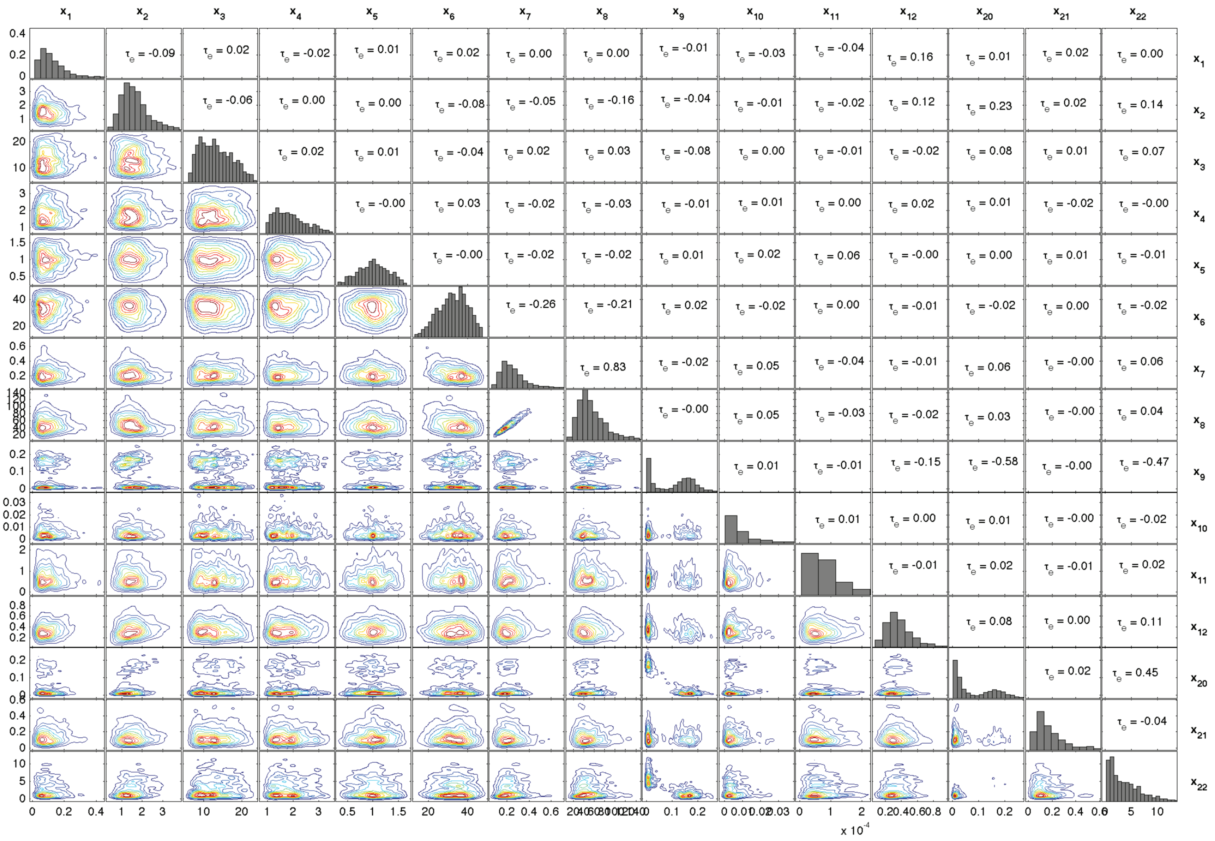

Figure 3: Scatterplot and Kendall's τs for the ICRP model based on the complete data runs

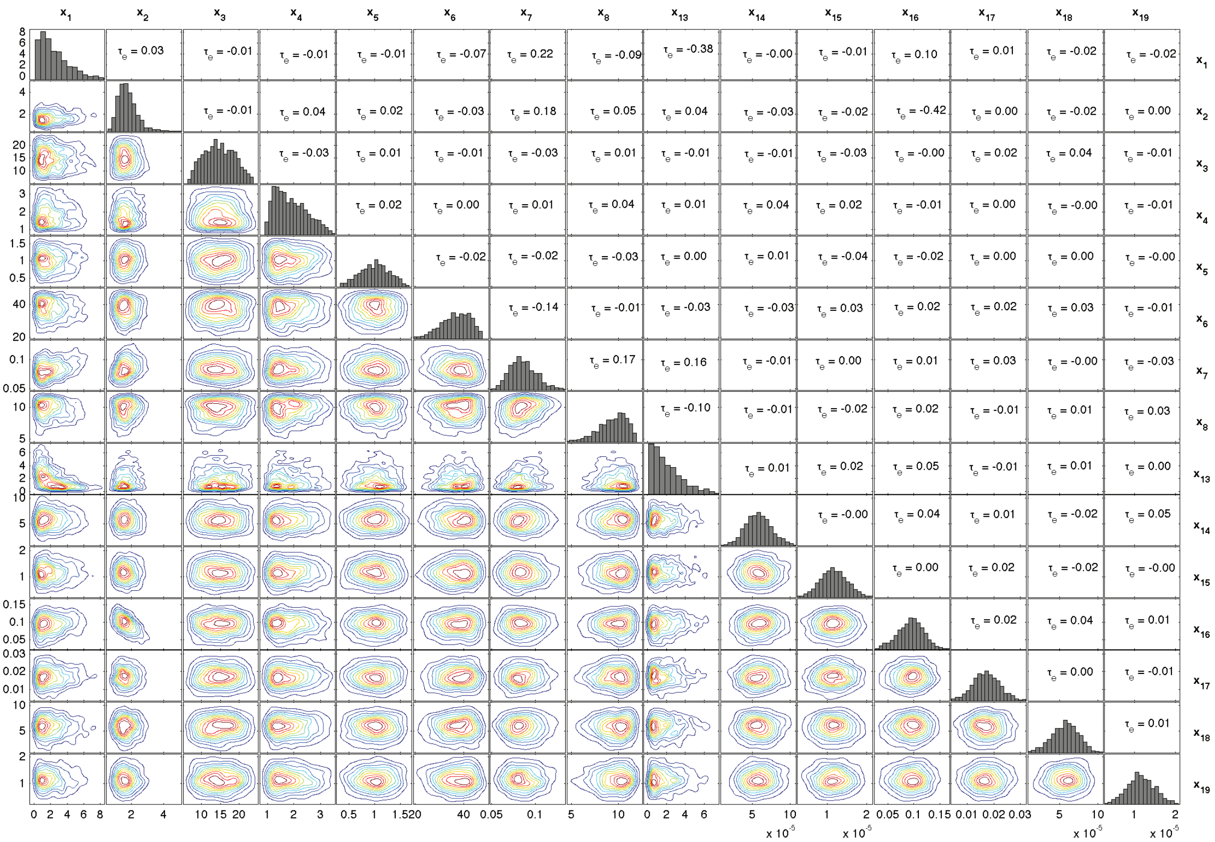

4.3 Identifiability analysis

We performed an identifiability analysis for the HMGU, extended HMGU, and ICRP model based on the according posterior distributions for each of the 16 investigations. The identifiability analysis was done as introduced by [Raue *et al.*, 2009] using profile likelihoods. For all investigations every model was clearly identifiable as can be seen in Figures 4, 5 and 6. Of course this is only true when using the posterior distribution and not the data likelihood for the profile likelihood, since otherwise alternative routes through the system could not be distinguished. The identifiability of the models induces a valid estimate of the maximum a posteriori estimate as well as the credible regions for the parameter estimates.

Figure 4: Profile likelihood for the HMGU model

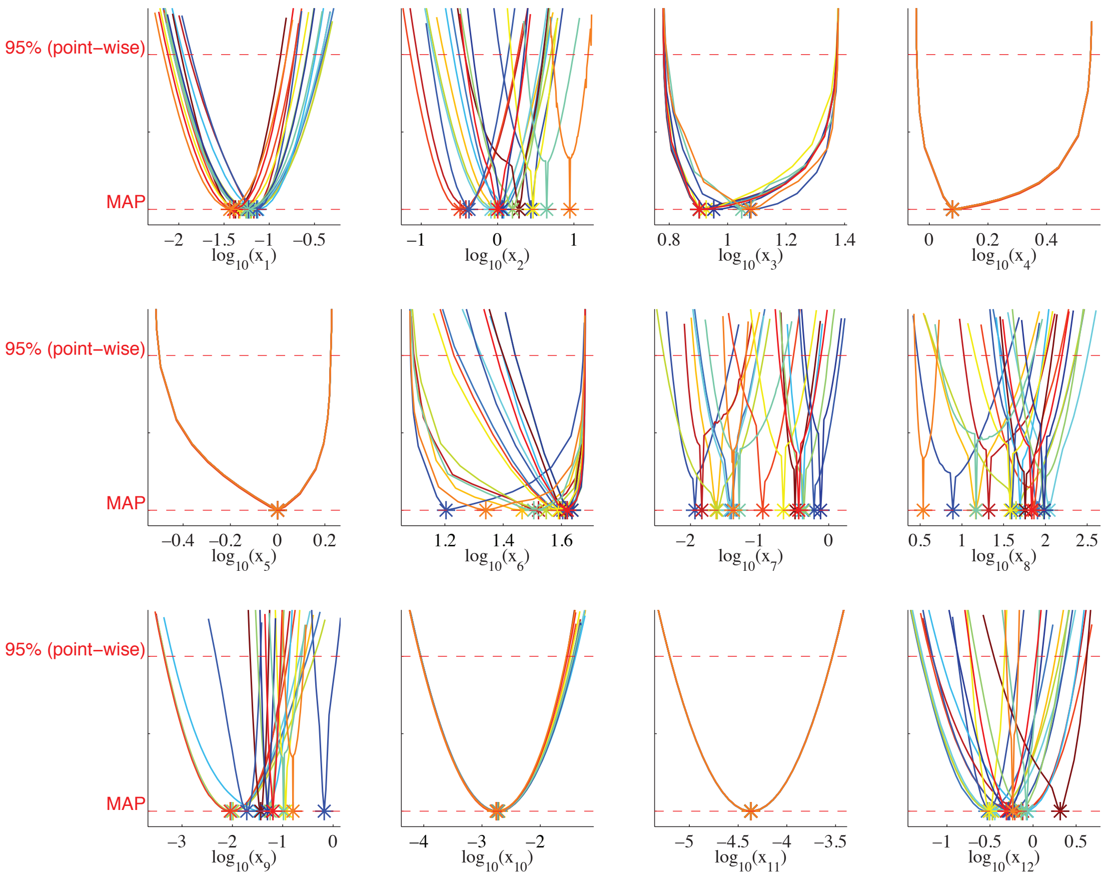

Figure 5: Profile likelihood for the extended HMGU model

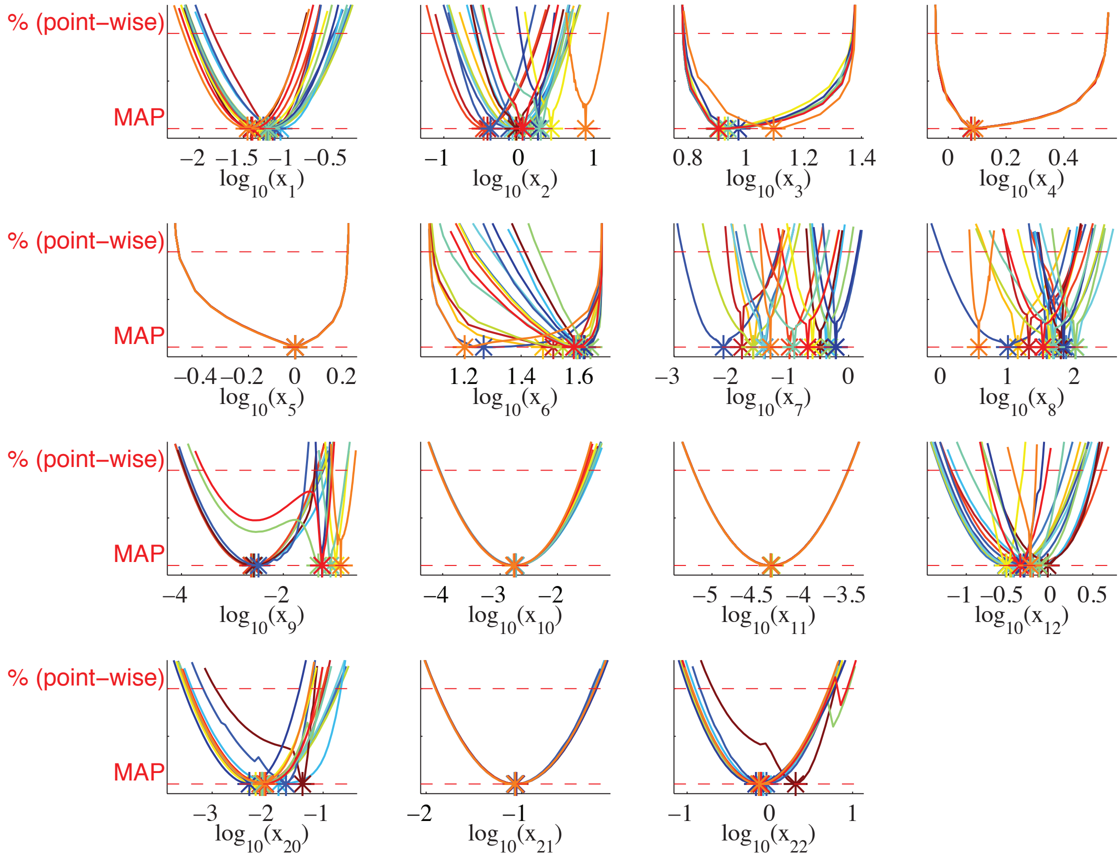

Figure 6: Profile likelihood for the ICRP model

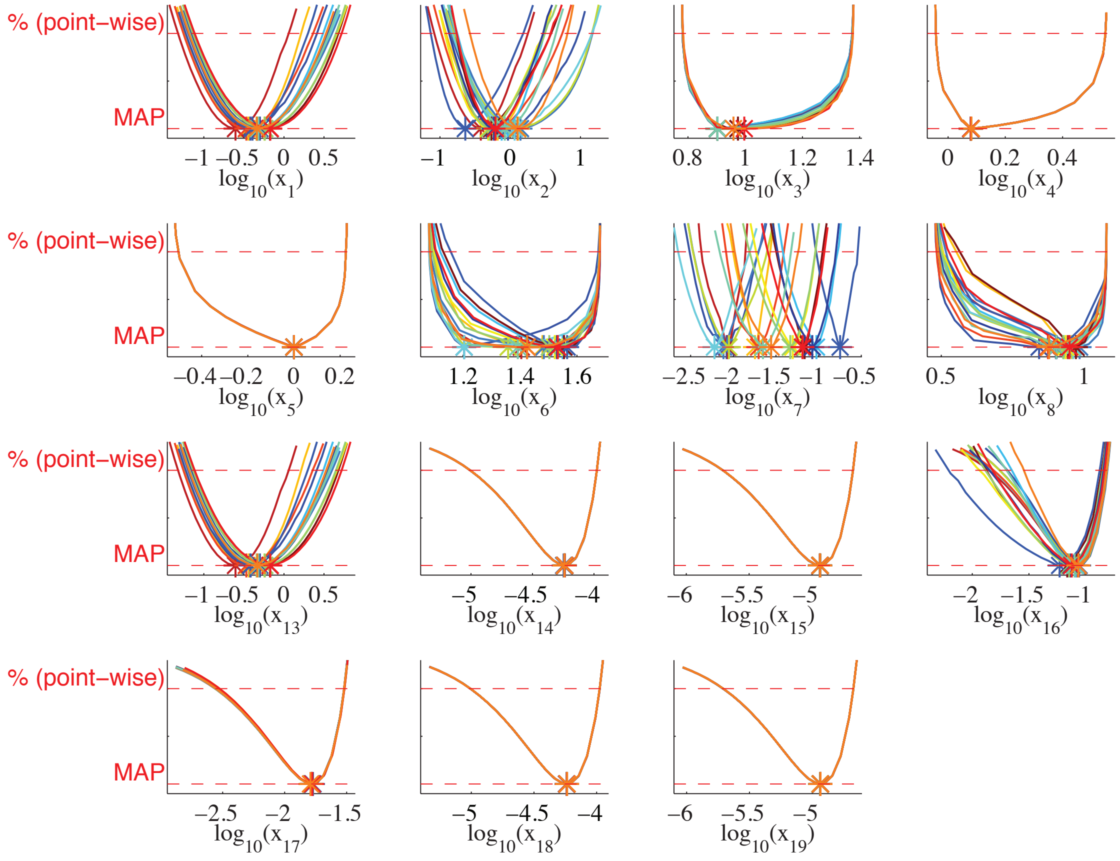

4.4 Time courses

We complement the time courses for the complete data model as seen in the main text with the time course plots for all the 16 investigations: For each investigation, all the time courses belonging to the posterior samples where calculated. We plot here the time courses for the transfer compartment and for the change in the urine compartment together with the corresponding data. For that, the black line is the pointwise median of the time courses, while the shaded area denotes the 90 % credible intervals of the time courses. The median and shaded areas represent the uncertainty in the parameters. Please note that neither the upper nor the lower boundary of the shaded area, nor the median need to be a time course belonging to a parameter sample. Shown in color are the data points for each investigation together with their errorbars, which display two standard deviations up and down. These standard deviations are NOT from the measurements, but the fitted standard deviations from the posterior.

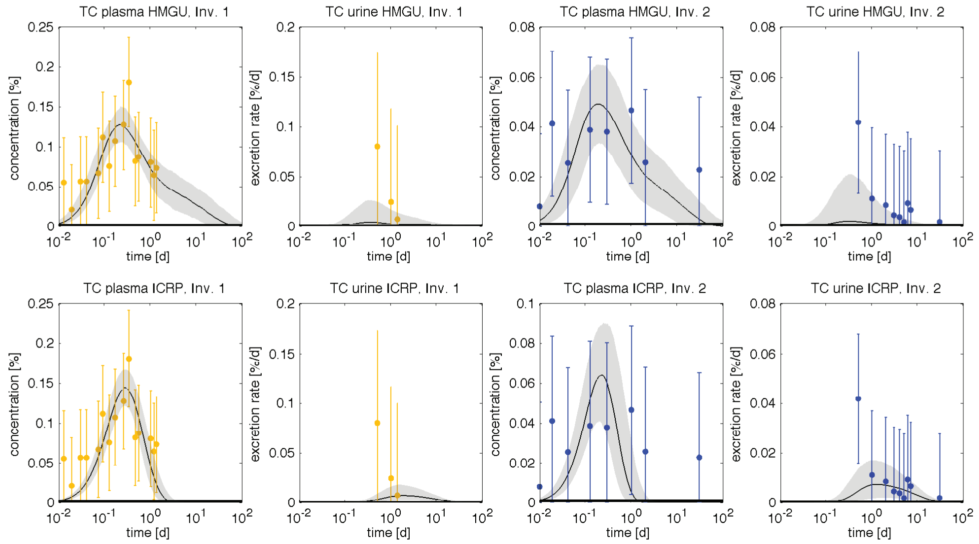

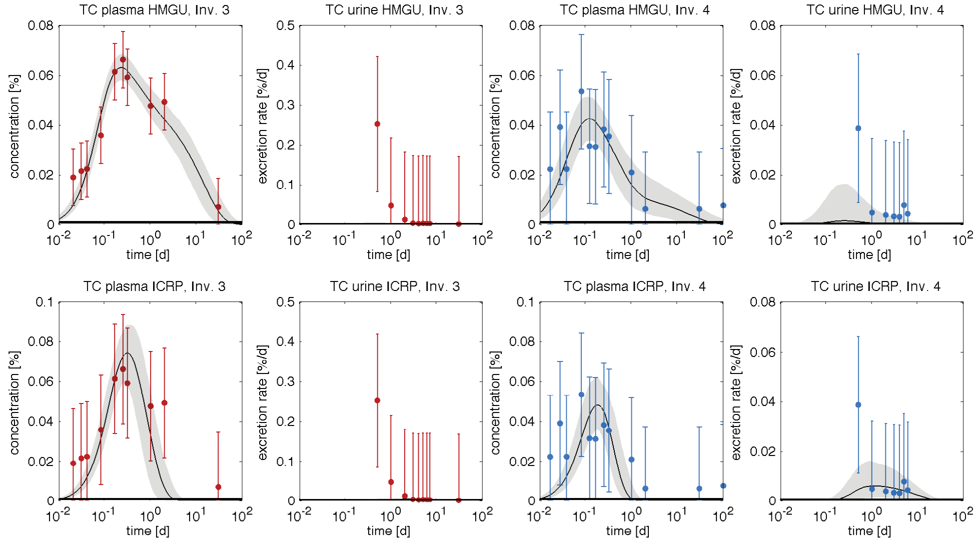

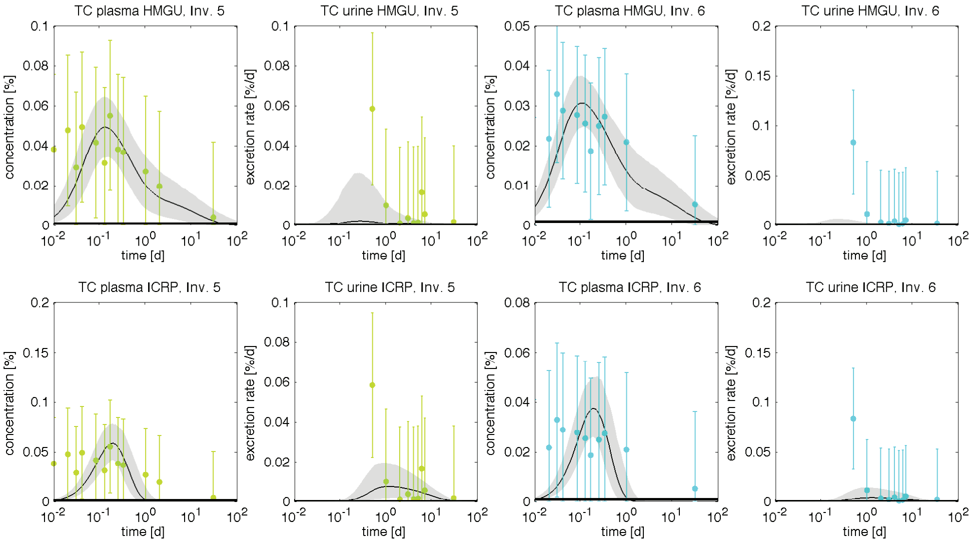
TC plasma HMGU, Inv. 5
TC urine HMGU, Inv. 5
TC plasma HMGU, Inv. 6
TC urine HMGU, Inv. 6
TC plasma ICRP, Inv. 5
TC urine ICRP, Inv. 5
TC plasma ICRP, Inv. 6
TC urine ICRP, Inv. 6
concentration [%]
excretion rate [%/d]
time [d]

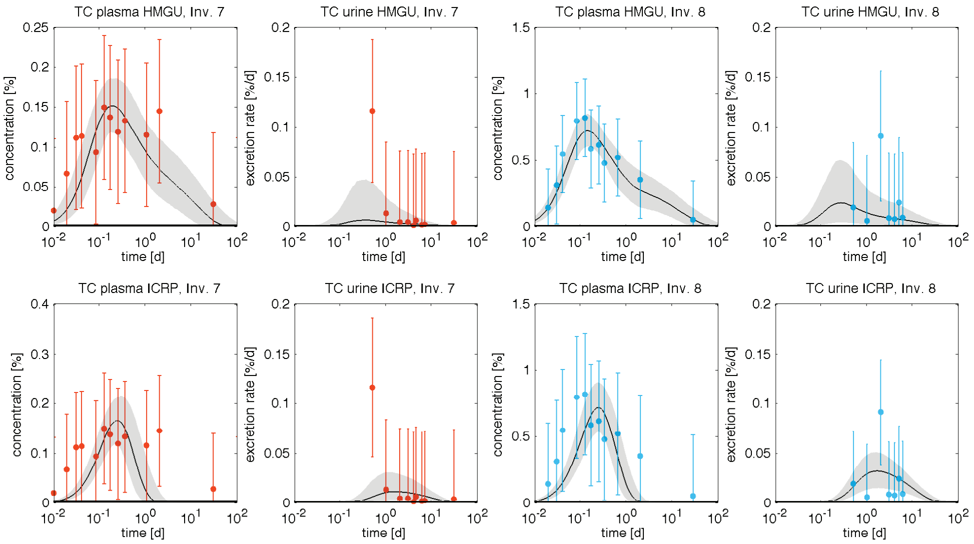
TC plasma HMGU, Inv. 7
TC urine HMGU, Inv. 7
TC plasma HMGU, Inv. 8
TC urine HMGU, Inv. 8
TC plasma ICRP, Inv. 7
TC urine ICRP, Inv. 7
TC plasma ICRP, Inv. 8
TC urine ICRP, Inv. 8
concentration [%]
excretion rate [%/d]
time [d]

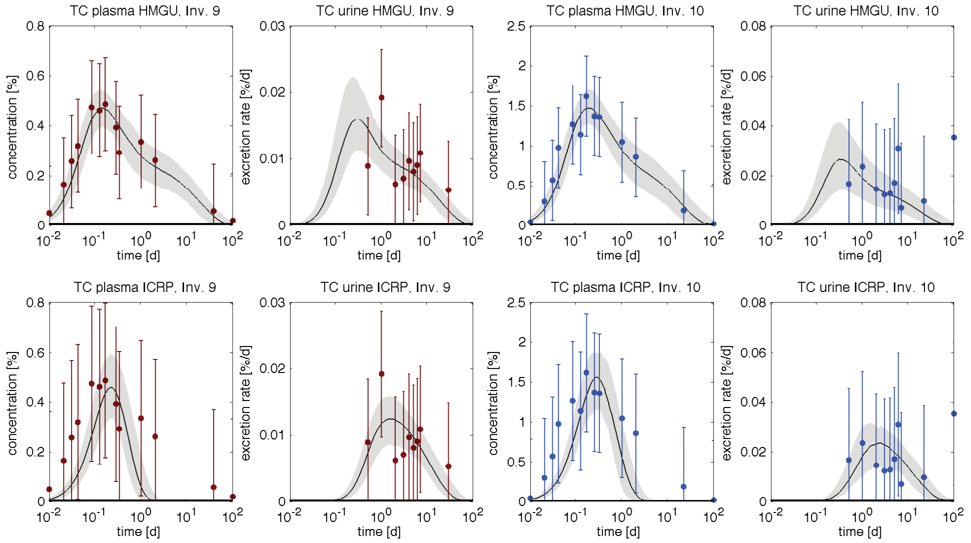
TC plasma HMGU, Inv. 9
TC urine HMGU, Inv. 9
TC plasma HMGU, Inv. 10
TC urine HMGU, Inv. 10
TC plasma ICRP, Inv. 9
TC urine ICRP, Inv. 9
TC plasma ICRP, Inv. 10
TC urine ICRP, Inv. 10
concentration [%]
excretion rate [%/d]
time [d]

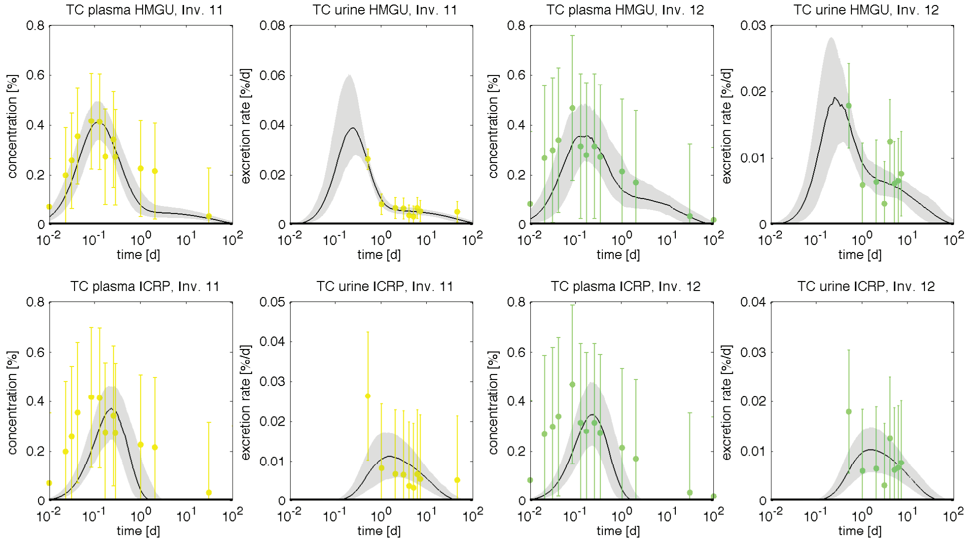
TC plasma HMGU, Inv. 11
TC urine HMGU, Inv. 11
TC plasma HMGU, Inv. 12
TC urine HMGU, Inv. 12
TC plasma ICRP, Inv. 11
TC urine ICRP, Inv. 11
TC plasma ICRP, Inv. 12
TC urine ICRP, Inv. 12
concentration [%]
excretion rate [%/d]
time [d]

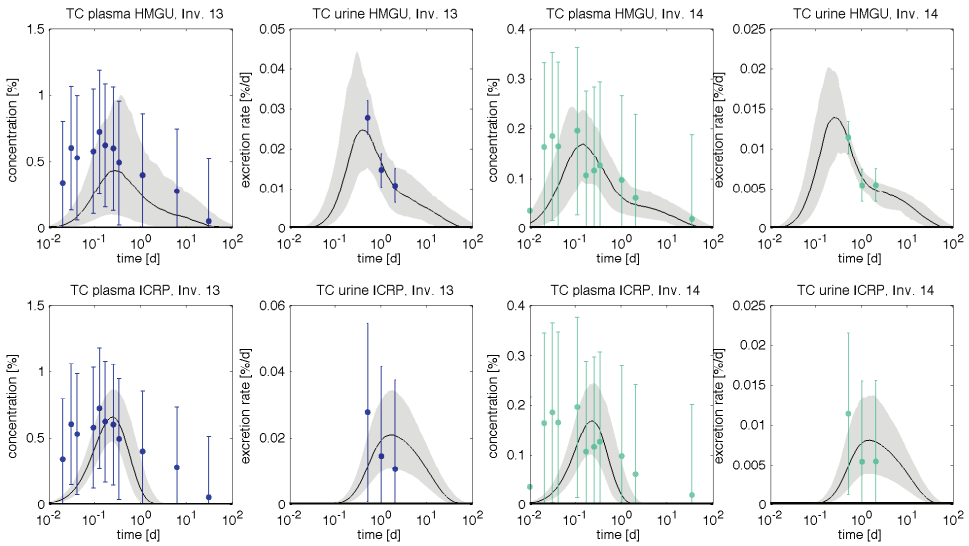
TC plasma HMGU, Inv. 13
TC urine HMGU, Inv. 13
TC plasma HMGU, Inv. 14
TC urine HMGU, Inv. 14
TC plasma ICRP, Inv. 13
TC urine ICRP, Inv. 13
TC plasma ICRP, Inv. 14
TC urine ICRP, Inv. 14
concentration [%]
excretion rate [%/d]
time [d]

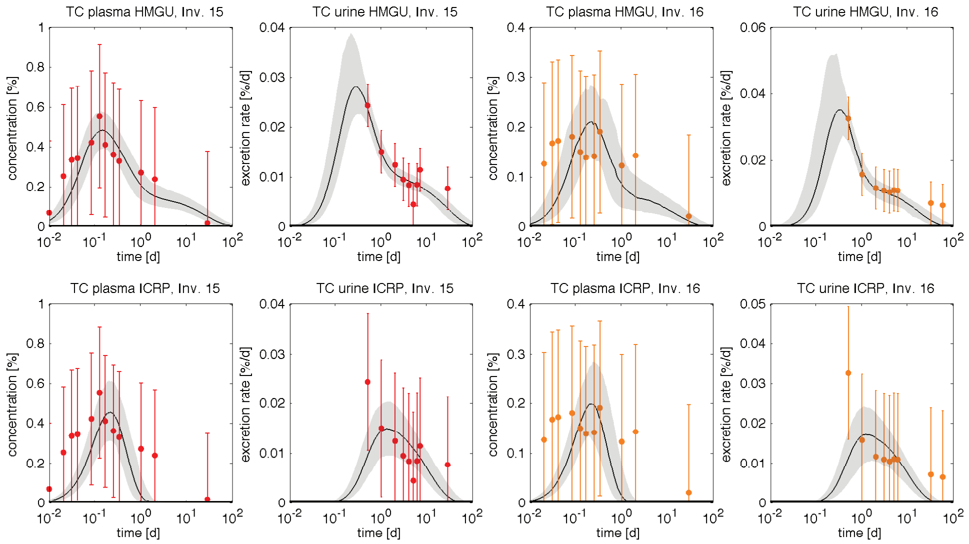
TC plasma HMGU, Inv. 15
TC urine HMGU, Inv. 15
TC plasma HMGU, Inv. 16
TC urine HMGU, Inv. 16
TC plasma ICRP, Inv. 15
TC urine ICRP, Inv. 15
TC plasma ICRP, Inv. 16
TC urine ICRP, Inv. 16
concentration [%]
excretion rate [%/d]
time [d]

References

[Greiter *et al.*, 2011] Greiter, M.B., Giussani, A., Höllriegl, V., Li, W.B., and Oeh, U. (2011) Human biokinetic data and a new compartmental model of zirconium – A tracer study with enriched stable isotopes, *Science of The Total Environment*, **409**, 3701-3710.

[ICRP, 1989] ICRP (1989) *Age-dependent Doses to Members of the Public from Intake of Radionuclides (Part 1). ICRP Publication 56*, Oxford: Pergamon Press; Ann. ICRP 20 (2).

[ICRP, 1993] ICRP (1993) *Age-dependent doses to members of the public from intake of radionuclides (Part 2). ICRP Publication 67*, Oxford: Pergamon Press; Ann. ICRP 23 (3-4).

[Jeffreys, 1998] Jeffreys, H. (1998) *Theory of probability*, Oxford: Oxford University Press.

[Johnson, 1994] Johnson, N.L., Kotz, S., and Balakrishnan, N. (1994) *Continuous univariate distributions*, New York: John Wiley\ & Sons.

[Li *et al.*, 2008] Li, W.B., Höllriegl, V., Roth, P., Oeh U. (2008) Influence of human biokinetics of strontium on internal ingestion dose of ^{90}Sr and absorbed dose of ^{89}Sr to organs and metastases. *Radiat. Environ. Bioph.*, **47**, 225-239.

[Li *et al.*, 2011a] Li, W.B, Greiter, M, Oeh, U., and Hoeschen, C (2011a) Reliability of a new biokinetic model of zirconium in internal dosimetry Part I, Parameter uncertainty analysis. *Health Phys.* **101(6)**: 660-676.

[Li *et al.*, 2011b] Li, W.B, Greiter, M, Oeh, U., and Hoeschen, C (2011b) Reliability of a new biokinetic model of zirconium in internal dosimetry Part II, Parameter sensitivity analysis. *Health Phys.* **101(6)**: 676-692.

[Raue *et al.*, 2009] Raue, A., Kreutz, C., Maiwald, T., Bachmann, J., Schilling, M., Klingmüller, U., and Timmer, J. (2009) Structural and practical identifiability analysis of partially observed dynamical models by exploiting the profile likelihood, *Bioinformatics*, **25**, 1923-1929.
